# Developing Institutional Capacity for Reproductive Health in Humanitarian Settings: A Descriptive Study

**DOI:** 10.1371/journal.pone.0137412

**Published:** 2015-09-02

**Authors:** Nguyen-Toan Tran, Angela Dawson, Janet Meyers, Sandra Krause, Carina Hickling, Inter-Agency Working Group (IAWG) on Reproductive Health in Crisis

**Affiliations:** 1 School of Public Health and Community Medicine, University of New South Wales, Sydney, Australia; 2 WHO Collaborating Centre, Faculty of Health, University of Technology, Sydney, Australia; 3 International Medical Corps, Washington, DC, United States of America; 4 Women’s Refugee Commission, New York, New York, United States of America; Centre for Geographic Medicine Research Coast, KENYA

## Abstract

**Introduction:**

Institutions play a central role in advancing the field of reproductive health in humanitarian settings (RHHS), yet little is known about organizational capacity to deliver RHHS and how this has developed over the past decade. This study aimed to document the current institutional experiences and capacities related to RHHS.

**Materials and Methods:**

Descriptive study using an online questionnaire tool.

**Results:**

Respondents represented 82 institutions from 48 countries, of which two-thirds originated from low-and middle-income countries. RHHS work was found not to be restricted to humanitarian agencies (25%), but was also embraced by development organizations (25%) and institutions with dual humanitarian and development mandates (50%). Agencies reported working with refugees (81%), internally-displaced (87%) and stateless persons (20%), in camp-based settings (78%), and in urban (83%) and rural settings (78%). Sixty-eight percent of represented institutions indicated having an RHHS-related policy, 79% an accountability mechanism including humanitarian work, and 90% formal partnerships with other institutions. Seventy-three percent reported routinely appointing RH focal points to ensure coordination of RHHS implementation. There was reported progress in RHHS-related disaster risk reduction (DRR), emergency management and coordination, delivery of the Minimum Initial Services Package (MISP) for RH, comprehensive RH services in post-crisis/recovery situations, gender mainstreaming, and community-based programming. Other reported institutional areas of work included capacity development, program delivery, advocacy/policy work, followed by research and donor activities. Except for abortion-related services, respondents cited improved efforts in advocacy, capacity development and technical support in their institutions for RHHS to address clinical services, including maternal and newborn health, sexual violence prevention and response, HIV prevention, management of sexually-transmitted infections, adolescent RH, and family planning. Approximately half of participants reported that their institutions had experienced an increase in dedicated budget and staff for RHHS, a fifth no change, and 1 in 10 a decrease. The Interagency RH Kits were reportedly the most commonly used supplies to support RHHS implementation.

**Conclusion:**

The results suggest overall growth in institutional capacity in RHHS over the past decade, indicating that the field has matured and expanded from crisis response to include RHHS into DRR and other elements of the emergency management cycle. It is critical to consolidate the progress to date, address gaps, and sustain momentum.

## Introduction

Approximately 51 million people are displaced by conflict and persecution [1]. Another nearly 22 million are internally displaced due to the sudden onset of a natural disaster [2]. As the majority of countries with the highest maternal and neonatal mortality are affected by crises [3,4], addressing the sexual and reproductive health needs and rights of affected women, men and adolescents is critical to ensuring their wellbeing, achieving the Millennium Development Goals (MDGs) 4,5, and 6, and the post-MDG agenda. Following the Balkan crisis and since the mid-1990s, reproductive health in humanitarian settings (RHHS) has been progressively mainstreamed into international standards [5]. The Inter-Agency Working Group on Reproductive Health in Crises (IAWG) was formed in 1995 with the mission to expand and strengthen access to quality RH services for people affected by conflicts and natural disasters. The IAWG established the global cornerstones for implementing RHHS: the Minimum Initial Service Package for Reproductive Health (MISP), the Inter-Agency Field Manual on Reproductive Health in Humanitarian Settings (IAFM), and the Inter-Agency Reproductive Health Kits [6–8].Over the past decade, the MISP has been included in key global health governance and funding processes that have given RHHS higher priority. These include the “cluster approach” put forward by the 2005 Humanitarian Reform to enhance leadership, accountability and predictability in humanitarian response [9], and the Central Emergency Response Fund (CERF) of which the MISP meets the life-saving criteria [10].

In 2004, the IAWG conducted a ten-year global evaluation of RHHS through a series of nested studies to identify gaps and constraints to inform resource planning and interventions for partners [11]. Findings showed that RH services were generally favorable for refugees in stable settings but were lacking for the internally displaced. The 2004 Global Evaluation studies included an assessment of organizational changes since 1995, focusing on agencies involved in RHHS. The major areas covered by that study included RH program components; organizational operations and policies; RH training and capacity building; technical assistance; resource tools; financial and staff resources; and collaboration between agencies. An important objective of the assessment was to document the level of commitment that organizations gave to the following five objectives of the MISP and the different types of work they undertook to support these objectives: 1) ensure effective coordination; 2) prevent sexual violence and provide clinical care for survivors; 3) reduce HIV transmission; 4) prevent excess maternal and neonatal morbidity and mortality; and 5) plan for comprehensive RH services [6]. In addition to the MISP, the study reviewed the components of comprehensive RH based on the 1999 version of the IAFM, which included safe motherhood, gender-based violence (GBV), sexually-transmitted infections (STIs), family planning (FP), and HIV [7]. Since 2004, the demonstrated trend of growth in capacity for technical expertise, collaboration, program activities, and institutionalization of the RHHS agenda has continued in many organizations, and the number of tools and resources to guide RHHS programming has increased over the last decade [12,13]. Greater attention has also been given to building national resilience to and preparedness for emergencies [14]. The aid architecture, as emphasized by the Paris Declaration and Accra Agenda of Action, has shifted toward country ownership and empowerment [15]. The new RHHS developments in disaster risk reduction (DRR) including emergency preparedness, early recovery, and protracted crises indicate IAWG’s growing efforts to bridging the humanitarian and development divide to ensure a more holistic, sustainable, and effective approach to health emergency management and health system strengthening efforts (emergency preparedness is a component of DRR; it is mentioned separately in this study as *DRR/emergency preparedness* as many countries have addressed it, but not the other components of DRR, which are referred to as *DRR/other components*) [14,16]. Anecdotal evidence suggests that more agencies have institutional policies related to RHHS, which also encompass broader entry points for RHHS, including DRR, emergency response, and during protracted crises or early recovery. However, little is known about the extent of institutional capacity and commitment to RHHS since the 2004 Global Evaluation. Therefore, as part of the 2013–2014 global evaluation of RHHS, the IAWG designed this study. It aims to gain insight into the overall state of RHHS over the past decade from an institutional perspective, by describing the capacity of government, non-government, United Nations, humanitarian, and development institutions to address RHHS.

## Materials and Methods

**Fig 1:**
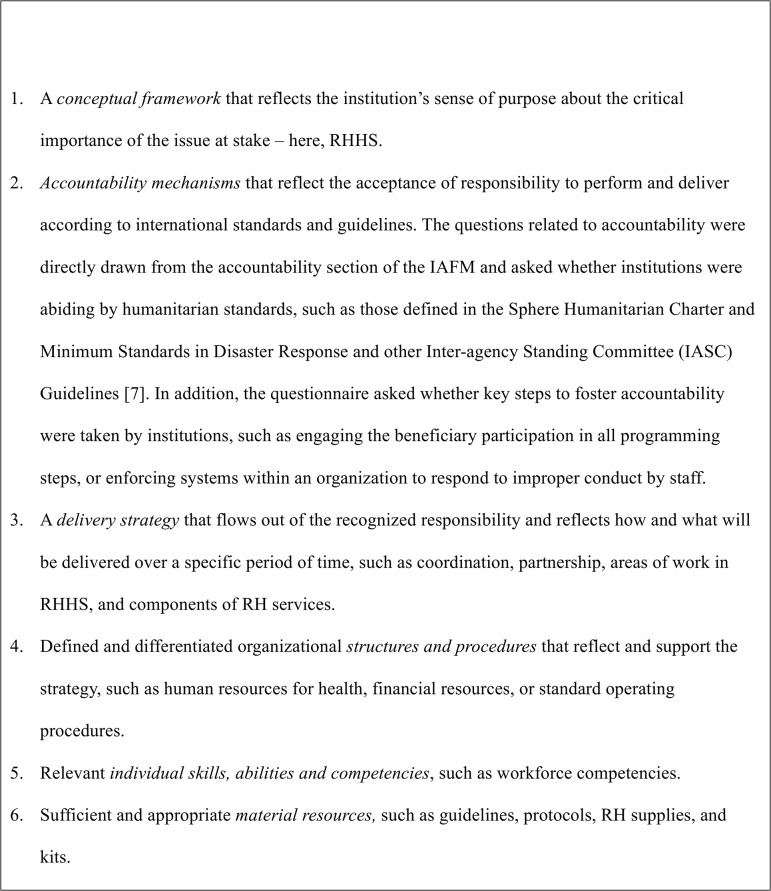
The six elements of the theoretical framework on institutional capacity applied in this research, based on Kaplan [17].

We undertook a descriptive study using a questionnaire tool with open and closed questions. The purpose of the questionnaire was to capture data about and gain insight into institutional capacity for RHHS along the emergency to development continuum including DRR, crisis response, early recovery and re-development, with trends in organizational changes over time. The questionnaire employed a structured theoretical framework based on Kaplan’s theoretical capacity building model [17]. Capacity, for the purpose of this study, is defined as the ability of an organization to function as a resilient, strategic, and autonomous entity [18]. The six elements of the capacity building model include institutional policy, accountability mechanisms, delivery strategy, and financial, human and technical resources related to RHHS (Fig 1). These components of institutional capacity can serve as proxy indicators of the overall state of RHHS and be helpful in illustrating how new approaches to addressing RHHS and institutional commitment to capacity development have been addressed at the field level. This study interpreted Kaplan’s conceptual framework as an institutional framework, which could comprise policies, guidelines, or other official supporting documents for RHHS. According to Kaplan, organizations are more likely to enable capacity building if they focus on developing an appropriate institutional framework that is driven by a concordant organizational attitude, vision, strategy, and supportive structures.

Therefore, the questionnaire tool was comprised of the following major components: purpose of the survey and consent, institutional characteristics, institutional policy, accountability mechanisms, program delivery strategy, structures and procedures, workforce competencies, and most useful material resources. The questionnaire tool was field-tested with selected institutions working at country and global level, and modifications were made based on this feedback. Ethical approval to carry out the study was obtained from the Faculty of Health of the University Technology, Sydney Australia (Nil/Neg Risk—UTS HREC 2013000209). The final questionnaire can be viewed online [19]. The English version was translated into French and back-translated into English to ensure veracity. Both English and French versions were administered online from April to August 2013 using the Smart-Survey software. A hard-copy of the questionnaire was made available to respondents with limited internet access. A quantitative descriptive design was adopted in order to gain a broad yet critical insight into the phenomenon of organizational capacity development in RHHS [20]. The target population comprised humanitarian institutions working in health or institutions working in RH and/or RHHS. Criterion sampling was used with organizations selected according to their membership of key groups involved in RHHS [21].Therefore, the questionnaire was sent to the listservs of the IAWG (n = 1292, representing 723 institutions), CORE Group (n = 1875, representing 354 institutions) [22] and Global Health Cluster (n = 118, representing 46 institutions) [23]. It was assumed that some institutions may be represented in more than one of the three listservs. But due to confidentiality concerns, it was not possible to obtain the detailed list of institutional names of each listerv to exclude double entries, and therefore calculate the total number of unique institutions that were invited to participate in the survey and determine the corresponding response rate. As this is a descriptive study design using an online questionnaire tool, our hope was to receive at least a similar number of responses from institutions as the number in the 2004 global evaluation (n = 30) and that participating institutions would not originate mostly from the global level as it was the case in 2004 but also come from country and field levels. We invited all organizations to complete a questionnaire and analysed the results using descriptive statistics. Based on the IAWG experience, institutions with the same name and working at different levels (national, regional, global) and in different countries often have different capacities and were assumed in this study to differ in their capacities and were handled as independent units of analysis. Therefore, each institution was invited per questionnaire instructions to select the most competent individual to represent their organization at the respective national, regional and international levels and participate in the research from the perspective of the level where they worked only. Consent was obtained from participants at the beginning of the questionnaire and respondents had the option to exit it at any time. Targeted follow-up by email was carried out to encourage responses from members of institutions in countries that had experienced humanitarian emergencies over the past decade but that had not yet answered the questionnaire. The data was extracted and transferred for analysis onto IBM SPSS Statistics 21. Data validation was done manually and through frequency checks and cross-tabulations. Descriptive statistical analysis was undertaken on all variables. Chi squared tests were undertaken on cross tabulations. Responses to the open questions were collated and summarised.

## Results

### Characteristics of respondents

**Fig 2:**
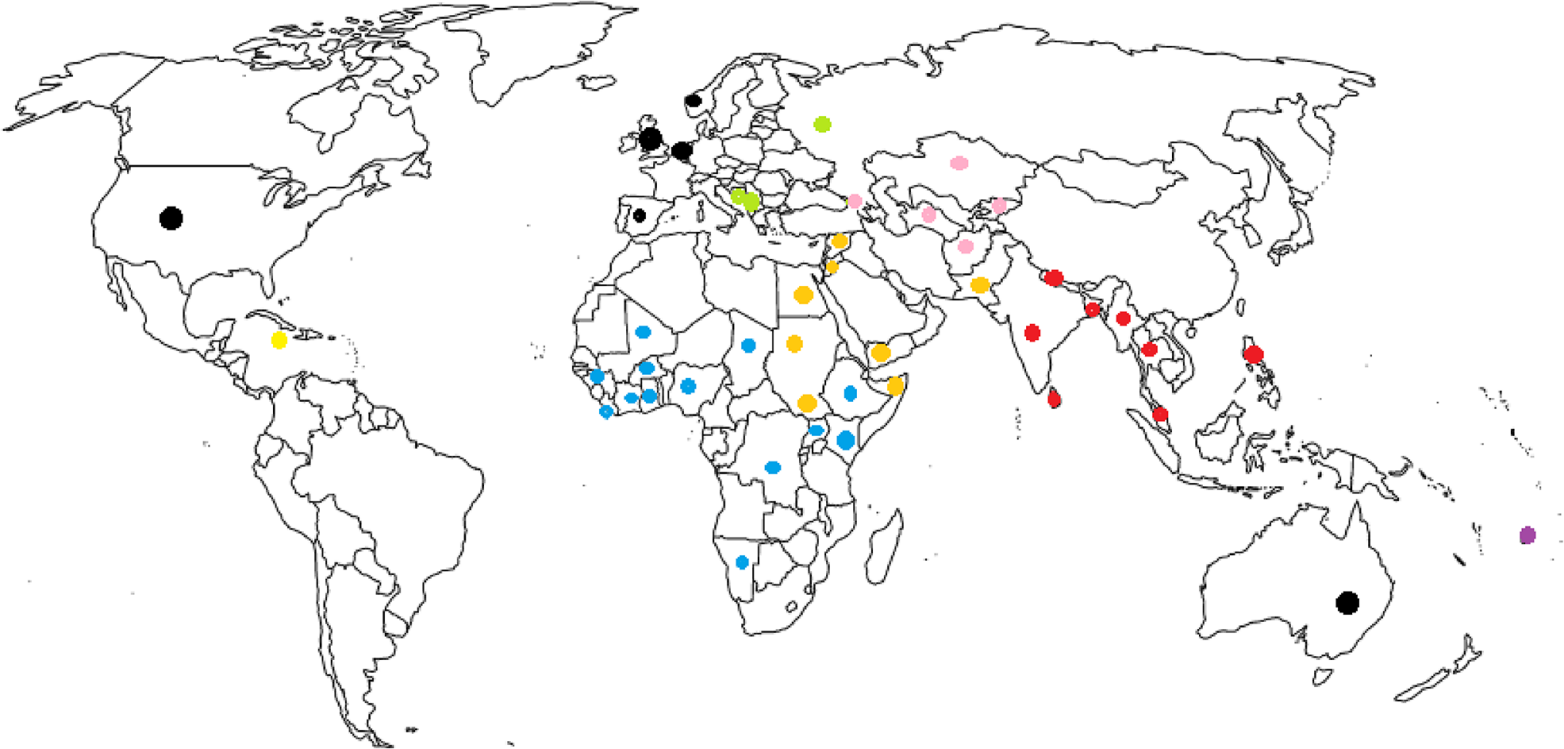
Map of the 48 countries from where the 82 institutional respondents originated.

Eighty-two institutions from 48 countries participated in the study (Fig 2), of which two thirds originated from low- and middle-income countries from Sub-Saharan Africa and Asia Pacific, and, to a lesser extent, the Middle East and Northern Africa, Eastern Europe and Central Asia, and Latin America and the Caribbean. Results indicate that most respondents held high-level management positions, as suggested by their job titles: head, director, coordinator, specialist, manager, representative, professor or assistant-professor, focal point, and others (Table 1). Fifty percent of institutions were NGOs, 34% United Nations agencies, 9% governmental institutions, and 7% academic institutions, with 71% of them working primarily at field and country levels. A large proportion of institutional respondents reported being members of the IAWG (85%). Chi-squared tests on cross-tabulations of variables in this section and all the other sections did not show any significance.

**Table 1:** Characteristics of respondents (n = 82) and nature of work.

**Distribution of respondents by geographical region (%)**		
	Australia, Western Europe countries, USA	33
	Sub-Saharan Africa	29
	Asia Pacific	24
	Middle East and Northern Africa	7
	Eastern Europe and Central Asia	5
	Latin America and Caribbean	1
**Types of institution (%)**		
	Non-governmental organisation	50
	United Nations	34
	Government	9
	Academia	7
**Membership to IAWG (%)**		
	Yes	85
	No	9
	Don't know	5
**Level of work (%)**		
	Primarily global level	22
	Primarily regional level	7
	Primarily field/country level	71
**Nature of work (%)**		
	Primarily humanitarian	25
	Primarily development	25
	Both humanitarian and development	50
**Types of settings where institutions work (%)**		
	Camp	78
	Rural	83
	Urban	83
**Crisis-affected populations institutions work with (%)**		
	Refugees	81
	Internally displaced persons (IDPs)	87
	Stateless	20
**Areas of reproductive health in humanitarian settings addressed by institutions (%)**		
	Capacity development (e.g. technical assistance)	93
	Program delivery (e.g. coordination, clinical services)	85
	Advocacy/policy	81
	Research	54
	Donor activities	39

### Nature of work

Twenty-five percent of institutional respondents reported the nature of their work to be primarily humanitarian, 25% primarily development, and 50% had an equal dual focus (Table 1). The majority of respondents reported that their institutions worked primarily at field or country level (71%), 7% at the regional level, and 22% at the global level, with 93% dealing with capacity development (e.g. technical assistance, training), 85% with program delivery (e.g. coordination, clinical delivery), 81% with advocacy and policy work, 54% with research, and 39% with donor activities. Respondents said their institutions operated not only in camp-based settings (78%), but also urban (83%) and rural settings (78%); with the affected populations they served being not just primarily refugees (81%), but also internally displaced persons (IDPs) (87%) and stateless persons (20%).

### Institutional framework

Two thirds of respondents (68%) reported their institution having an RHHS-related policy or guideline, or other official support document, such as a Board mandate, 23% reported not having such a document, and the remainder did not know. Although most respondents from institutions with a humanitarian focus reported having a RHHS-related document (82%), respondents from development institutions also indicated that they were supported by an RH policy (61%). Emergency response was the most commonly reported policy content (91%), followed by DRR/emergency preparedness (72%), recovery (63%), DRR/other components (42%), and research policies (25%).

### Accountability

Seventy-nine percent of respondents reported that their institution had an overall accountability mechanism that also covered humanitarian work, 13% did not have one and the remainder did not know. A vast majority of respondents stated that their institutions had policies and systems in place to comply with global standards, such as the IASC Guidelines for Gender-based Violence Interventions in Humanitarian Assistance and others [5,7,24–31]. In addition, respondents noted that their institutions had mechanisms in place to ensure that a number of steps were taken with regard to accountability. These include: RH indicators collected as part of the institutional health information system and/or monitoring and evaluation system (86%); engagement of beneficiaries in all programming steps—assessing, planning, implementing and monitoring the project (70%); compliance with systems within the organization to respond to improper conduct by staff (69%); the establishment of ongoing communication with affected populations about the institution and its project plans and work (69%); and the institution of mechanisms for beneficiaries to contact organizational representatives, lodge complaints and seek redress (49%).

### Program delivery strategies

#### Partnerships and coordination

A majority (90%) of respondents reported that their institution participates in RH coordination mechanisms or working groups and 73% reported that their institutions routinely invested in an RH focal point or officer to ensure effective coordination of the MISP. The leading coordinating institutions were the UN (85%), government agencies (62%), and NGOs (58%). Institutions reported to hold formal partnerships with the following other institutions working in RHHS: UN agencies (71%), government institutions (62%), international NGO (62%), national NGO (56%), community-based organizations (41%), and private sector (15%).

#### Areas of work

**Fig 3:**
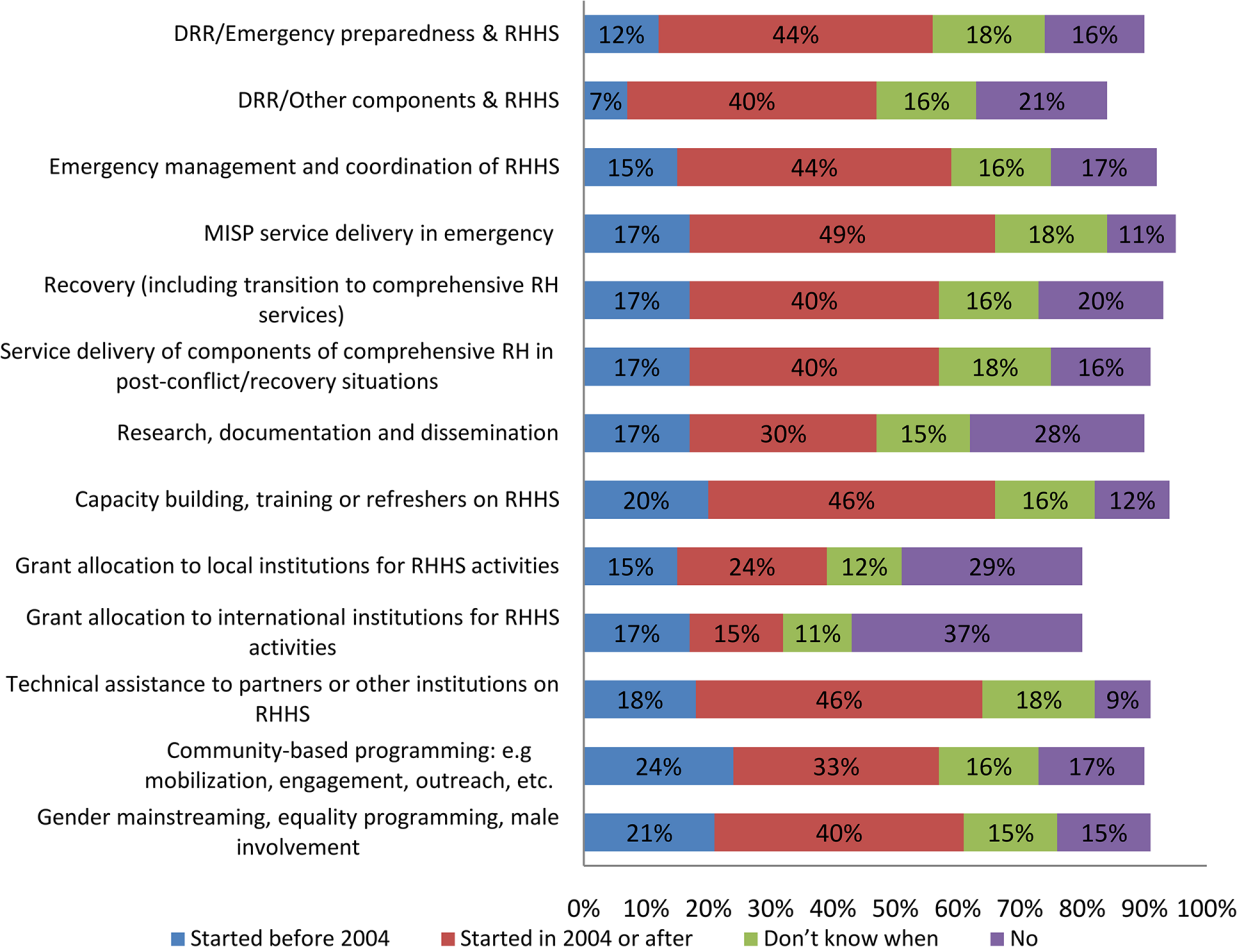
Areas of work in reproductive health in humanitarian settings addressed by institutions before and since 2004 (n = 82).

Results indicate that from 2004, almost all of the areas of work related to RHHS were reported by participants to have experienced an increase in institutional coverage, with the most activity occurring in relation to MISP implementation, capacity development, and technical assistance, with approximately half of the institutions having started such activities in 2004 or after (Fig 3). Growth was also reported in RHHS-related DRR/emergency preparedness, emergency management and coordination, delivery of comprehensive RH services in post-crisis/recovery situations, recovery, gender mainstreaming, DRR/other components, advocacy and policy work, and community-based programming (most notably in the areas of FP, GBV, and maternal and newborn health).

#### RH services

**Fig 4:**
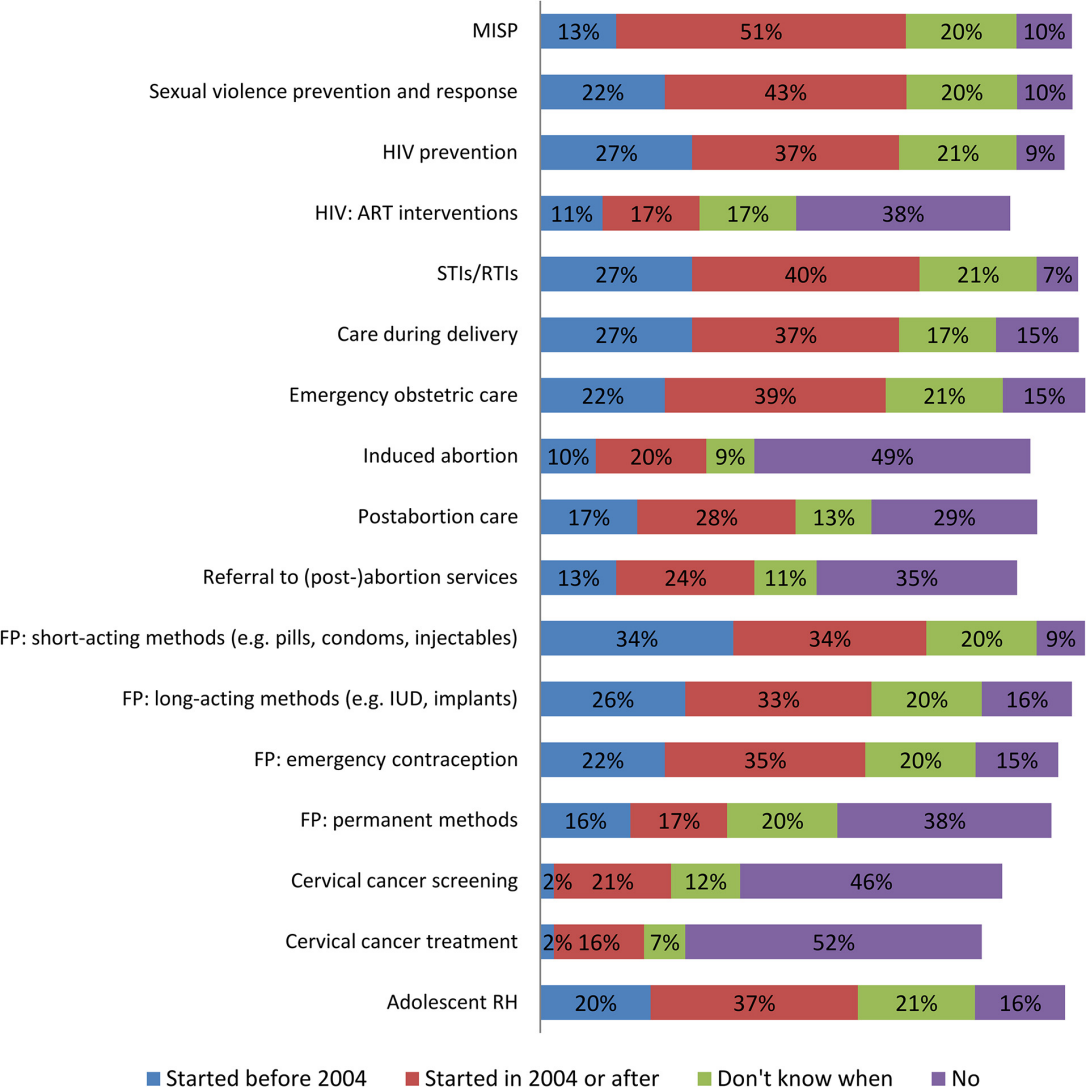
Clinical reproductive health in humanitarian settings services addressed by institutions before and since 2004 (n = 82).

The results show increased activity over the past decade in almost all clinical areas of work in the institutions from where participants were drawn (Fig 4). These clinical areas were not exclusive to service provision and could involve guideline development, service delivery, technical assistance, training, advocacy, or research. Increases in the institutional coverage of a number of areas were noted by respondents including MISP-related services and some comprehensive RH services: maternal and newborn health, sexual violence prevention and response and broader GBV prevention, HIV prevention, management of sexually-transmitted infections (STIs) or reproductive tract infections (RTIs), adolescent RH, and FP, including emergency contraception. Results indicate that respondents felt that institutions had increased the delivery of HIV care and support including ARV interventions since 2004, but overall, the findings show that this area of activity had less institutional coverage than other components of RH care, despite the fact that the MISP recommends the provision of ARVs for individuals already taking them and for PMTCT. According to respondents, institutions were less active in terms of abortion-related services, which are part of the MISP, cervical cancer screening and treatment, and permanent methods of FP, which are components of comprehensive RH services. With regard to abortion-related services, half of all respondents (49%) reported that their institutions did not conduct activities related to induced abortion, and approximately a third did not address post-abortion care (29%), or referral to safe abortion or post-abortion services (35%). As for FP, institutions reported providing not only short-term methods (88%, e.g. pills, condoms, injectables), but also emergency contraception (77%), long-acting FP methods (79%), and postpartum FP (70%). Permanent FP methods were reportedly addressed by 53% of institutions. With regard to cervical cancer screening, approximately half of respondents said their organizations did not undertake screening (46%) or provide treatment (52%).

### Financial resources

**Fig 5:**
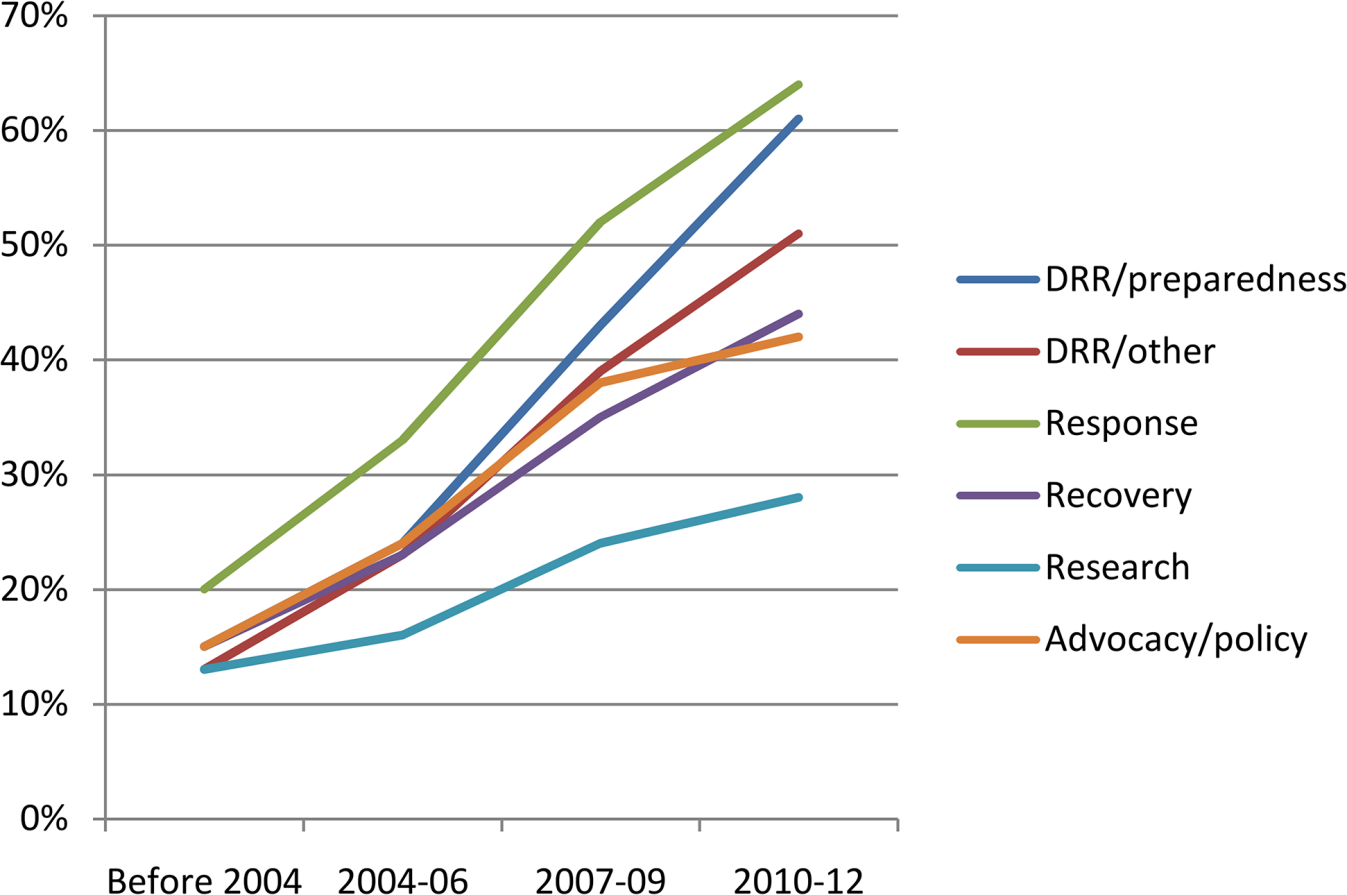
Proportion of institutions with dedicated budget for areas of work related to reproductive health in humanitarian settings by time period (n = 82).

The 2004 Global Evaluation reported an increase in the overall organizational expenditure for RHHS over the previous decade though it did not reflect an overall global increase in funding for RHHS. The 2013 results show that the overall trend in organizational expenditure has continued after 2004 for half of the respondents (49%) and concerned DRR, response, recovery, advocacy/policy work, and to a lesser extent research, while 20% did not report substantial change and 13% reported a decrease (Fig 5).

### Human resources

**Fig 6:**
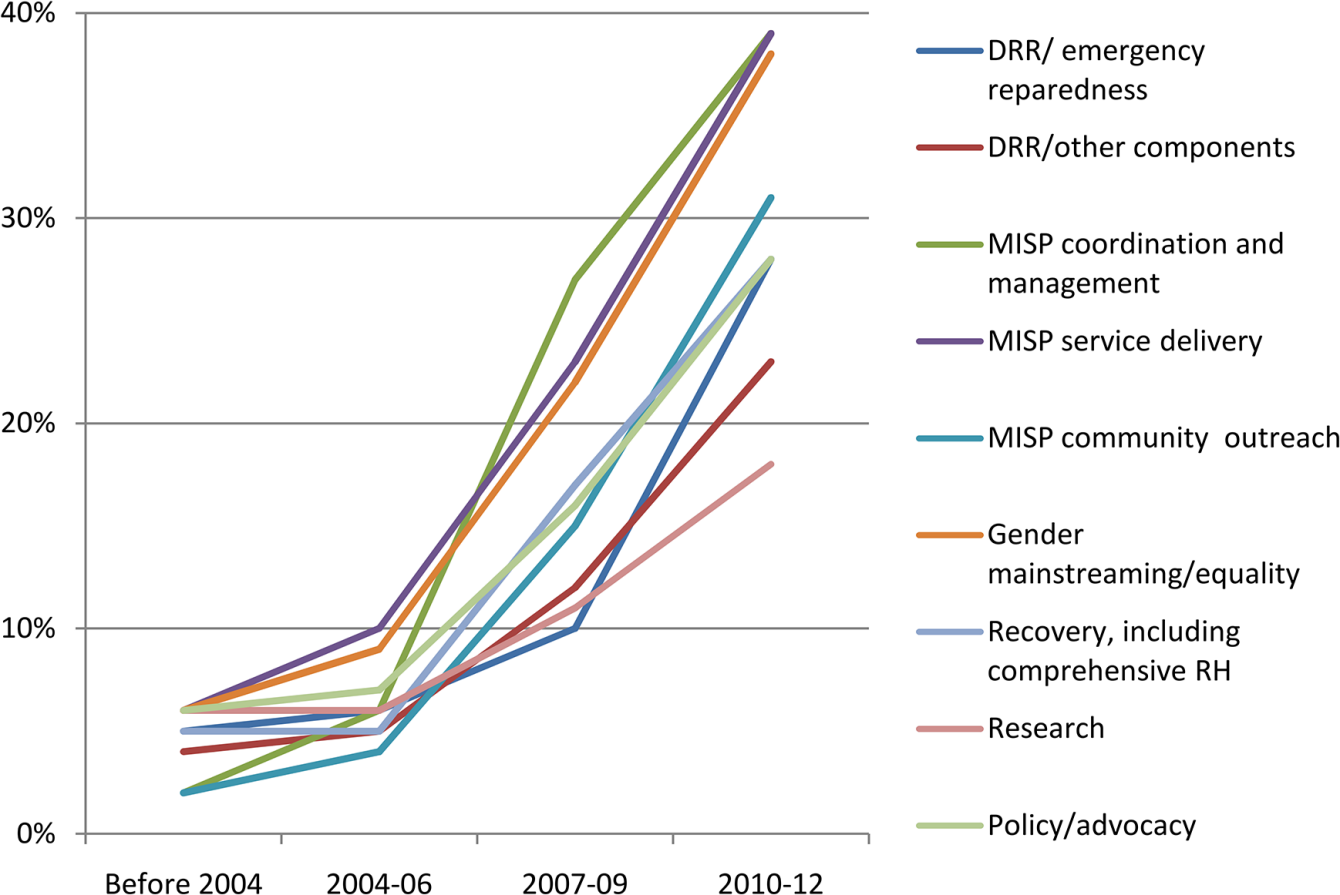
Proportion of institutions reporting high-level workforce competencies in different areas of reproductive health in humanitarian settings by time period (n = 82).

Although 22% of respondents reported no change in the number of dedicated staff to RHHS over the past decade, and 15% a decrease, 50% reported an increase, with a growing number of staff having moderate to high levels of competencies (Fig 6). These competencies include the MISP, gender-mainstreaming and other components of the emergency management cycle such as DRR and recovery.

### Guidance

There is a wealth of materials related to RHHS [12,13]. Among the 21 publications that institutional respondents had to rank, the landmark IAFM stood out as the most useful publication. This document and the other top five most useful publications support the field implementation of the MISP [6,7,8,32,33]. Except for the Sphere Handbook [5], the publications ranked six to ten focus on a specific topic, including GBV and adolescent RH [24,34,35,45]. With regard to guidance that institutional respondents wished that the IAWG develops to support their RHHS work and workforce in the next five years, monitoring and evaluation, research, documentation and dissemination on RHHS came on top, along with DRR/emergency preparedness, advocacy and policy work, and emergency management and coordination.

### Commodities

Respondents noted that institutions concerned with service delivery and/or the procurement of RHHS commodities reported using the Interagency Reproductive Health Kits (65%), which are designed and regularly updated by the IAWG and directly support MISP implementation [8]; local or regional supply chains (51%); their institution’s supply chain (34%); and the Interagency Emergency Health Kits (23%), which include some RH supplies, but not all that are needed to support MISP implementation. In the previous five years, respondents indicated experiencing the following challenges with regard to the procurement of RH supplies: delay in obtaining or distribution of Interagency RH Kits (81%), difficulty in sourcing quality RH supplies (56%), delay in identifying suppliers for RH commodities (49%), and stock out of RH supplies (21%).

## Discussion

The results suggest an overall picture of growth in institutional capacity in RHHS over the past ten years. This progress is illustrated across a number of areas, including: involvement of institutions that are not primarily humanitarian; inclusion of other beneficiaries and in new contexts, such as IDPs, stateless persons, and urban settings; establishing institutional frameworks for RHHS; setting up accountability mechanisms supporting RHHS; and working through partnerships and broadening program delivery strategies to integrate DRR and the emergency management cycle (Table 2). Additional areas include: improving the quality and comprehensiveness of clinical services, although key services are still not sufficiently prioritized, such as abortion-related services; and reinforcing investments in dedicated human and financial resources for RHHS. However, the results of this research need to be carefully interpreted as they only allow us to glean some insight into self-reported organizational capacity, which does not reflect quality of or access to services on the ground.

**Table 2:** Institutional capacity for RHHS in the 2004 and 2013 global evaluations: summary of key findings.

	2004 global evaluation	2013 global evaluation
**Participating institutions**	Mostly humanitarian institutions from the “global North” (n = 30).	Humanitarian and development institutions with a majority of them based at field/country level (n = 82).
**Institutional policies supporting reproductive health in RHHS**	Less than half of institutions (43%).	A majority of institutions (68%).
**Accountability mechanisms related to RHHS**	Not assessed.	A majority of institutions (79%).
**Target populations**	Mainly refugees.	Refugees, internally displaced persons, stateless persons.
**Main services focused upon**	More than half reported a focus on safe motherhood including emergency obstetric care, GBV, HIV, STIs, FP; and youth programs.	MISP within a wider disaster risk reduction and emergency preparedness framework; STIs and adolescent RH.
**Main gaps in services**	Half or less reported to focus on MISP, female genital mutilation, sexual violence including sexual exploitation and abuse, domestic violence, ART including PMTCT, and emergency contraception.	Post-abortion care and comprehensive abortion care services, permanent methods of contraception, and cervical cancer screening and treatment.
**Institutional budget for RHHS**	Overall growing investment.	Overall growing investment.
**Institutional human resources for RHHS**	Overall growing investment (86% of respondents reported increase).	Continued investment (50% of respondents reported increase, 22% no change, and 15% reported a decrease).

ART: antiretroviral therapy, FP: family planning, GBV: gender-based violence, MISP: minimum initial service package for reproductive health, PMTCT: prevention of mother-to-child transmission of HIV, RHHS: reproductive health in humanitarian settings, STIs: sexually-transmitted infections.

It is worth noting the global reach of the study although only a few responses came from the Middle-East and North Africa region. This is less than what would be expected in light of the several crises in that region over the past years, and may be due to the fact that the questionnaire tool was only available in English and French. The high proportion of institutional respondents with IAWG membership may reflect the IAWG’s successful outreach and scaling-up efforts over the past decade to strengthen country and regional capacity and ownership in RHHS.

With regard to the nature of work of institutions, results suggest that RHHS is no longer a sole domain for humanitarian institutions. This may reflect a shift in the landscape of RHHS where increased emphasis has been placed on a holistic approach to addressing RHHS within the emergency management cycle in which humanitarian and development institutions alike have a joint role to play [14]. The inclusion of programs involving not only refugees, but also IDPs and stateless persons contrasts with the 2004 results, which indicated that services for IDPs were severely lacking, and where there was no mention of stateless persons.

Adopting an institutional framework for RHHS can serve as a roadmap and help catalyze the prioritization and implementation of RHHS activities within an organization. The findings indicate that over the past decade, and in particular during the 2010–2012 period, there has been an increase in the number of institutions, either primarily humanitarian or development, supported by an RHHS-related policy, guideline or other official support documents, such as a Board mandate. This suggests progress in institutionalizing RHHS considering that in 1995, when the IAWG was formed, only one institution had an RHHS policy, and in 2004, 43% of those surveyed had an RHHS policy [11].

There has been a movement in the humanitarian community towards ensuring accountability to recipients of assistance, as illustrated by the development of international standards, especially the Inter-Agency Standing Committee (IASC) guidelines and the Sphere Handbook and other major principles of accountability. Our results suggest that this movement has also been reaching many institutions working in RHHS, with a majority of them having mechanisms in place to abide by such principles. Institutions with a presence at the field and regional level may rely on the accountability mechanisms established by their headquarters; the extent to which these mechanisms actually inform field-level programming require in-depth assessments that were not part of the scope of this study. Therefore, further research is needed to examine the nature and extent of accountability mechanisms adopted by institutions and how they actually influence program implementation in the field.

In terms of program delivery strategies, results indicate that partnerships and coordination remain critical, reflecting the findings of the 2004 survey where formal partnerships and inter-agency coordination were found to be key elements. As for the areas of work, institutions appear to keep an emphasis on core activities, such as MISP implementation, capacity development, and technical assistance while results also suggest the development of work in emerging areas, such as DRR. Increased commitment to implementing the MISP is also reflected in the MISP assessment and funding studies of the 2012–2014 IAWG global evaluation [36, 37].

Service delivery appears to be hampered by challenges with timely access to RH supplies which is also consistent with the IAWG field study in three humanitarian settings [38]. Respondents’ views on their institutional capacity to make other FP methods, such as emergency contraception, long-acting and postpartum methods, increasingly available in RHHS programs are encouraging and show increased activity compared with the results of the 2004 Global Evaluation, which highlighted gaps related to the availability of methods [11]. However, other global evaluation studies show emergency contraception and long-acting FP methods a gap [38], which may reflect agencies focus in these areas on advocacy and capacity development with a time-lag between what agencies are aiming to implement and what they are actually managing to do. As expected, components of comprehensive RH that are more complex in terms of programming or that were recently included in the IAFM, such as safe abortion care, ARV provision, PMTCT, cervical cancer screening and treatment, and permanent FP methods, received less reported institutional coverage and are consistent with other global evaluation studies [36, 38]. These findings highlight the needs for the IAWG to develop a well-planned and well-resourced strategy to tackle these gaps. In particular, emphasis needs to be given to the delivery of safe abortion care as part of MISP implementation. The inclusion of a new Comprehensive Abortion Care chapter in the 2010 revision of the IAFM is a remarkable step forward. Other RH services that were found to receive less focus, such as the provision of ARVs for continuing users or for PMTCT, cervical cancer screening and treatment, and permanent FP methods, also need to be addressed. Overall, however, these findings suggest progress towards building institutional capacity with regard to RHHS services.

The positive development of institutional capacity globally is also suggested by the overall trend in organization expenditure which has continued after 2004 or did not change substantially for a majority of institutions. This is a positive finding in spite of the global financial crisis of 2008 and its aftermath, and demonstrates the importance and commitment given to RHHS by institutions, including donors [39]. The fact that almost three quarters of institutions reported routinely investing in an RH focal point or officer is also encouraging since past MISP implementation assessments have frequently highlighted weak coordination that was partially due to the lack of a dedicated RH focal point [40]. The 2004 Global Evaluation further pointed to the lack of agreement among respondents on the definition of RH focal point. This situation has now been clarified and the IAWG has terms of reference for the RH focal point that were added to the 2010 revised version of the IAFM to help implementing partners address this critical step [41]. Since the mid-2000s, several global initiatives have been established that may have contributed to the overall workforce strengthening in RHHS, including: the RAISE Initiative [42], the SPRINT Initiative [43], several training courses on sexual violence [44–46], and the MISP Distance Learning Module, which more than 4,000 people have completed online since 2006 [6,47].

With regard to guidance, the IAFM stood out as the most useful publication along with the ones supporting the field implementation of the MISP. The fact that guidance materials related to managing GBV and adolescent RH were also ranked highly illustrate the importance given to addressing these critical gaps. Adolescent RH is a cross-cutting but often overlooked theme in RHHS [48]. Partners should take note of the wealth of existing materials related to RHHS, as designing guidance and training resource packages to develop workforce capacity is resource-consuming and should be thoroughly planned and evaluated from design to implementation phases.

Results suggest that the Interagency RH Kits remain the most commonly used source of supplies (along with other local, regional, and global sources), but that the timely distribution of the kits in-country remained a major challenge. Although multiple face-to-face logistics training workshops were conducted over the years by IAWG partners, further studies are needed to examine and address the commodity barriers and challenges reported by respondents.

### Study limitations

The results of this research provide a snapshot of self-reported organizational capacity development among the participating institutions and does not equal to action on the ground or reflect quality of services. Non-probability sampling means that the results of this study are not representative and reflect the views and experiences of those who participated. Respondents may have had more interest in RHHS than non-respondents which may have led to a lack of perspective from organizations without this focus. However this in itself may indicate that more work is to be done to further engage organizations in RHHS. Further, agency representatives may have reported intentions given their engagement in internal dialogue or coordinated efforts, and not necessarily service delivery that had actually begun.

The responses exclude the inputs from those unable to complete the questionnaire in English or French and do not represent all humanitarian emergencies and RHHS interventions in all communities covered by participating institutions. However, the study suggests capacity growth across a number of institutions. Further research is needed to examine whether this reflects trends across all institutions and the relationship between factors that help to facilitate institutional capacity development. The reported changes in organizational capacity over time are general descriptions relying on institutional memory, which participants may have had difficulty recalling. To follow trends in real time requires repeated representative cross sectional surveys at different points in time, which can be expensive and time-consuming. The current findings will also be useful as a reference for assessing progress in institutional capacity when future evaluations will take place, including the next Global Evaluation that will likely occur again in ten years.

## Conclusions

**Fig 7:**
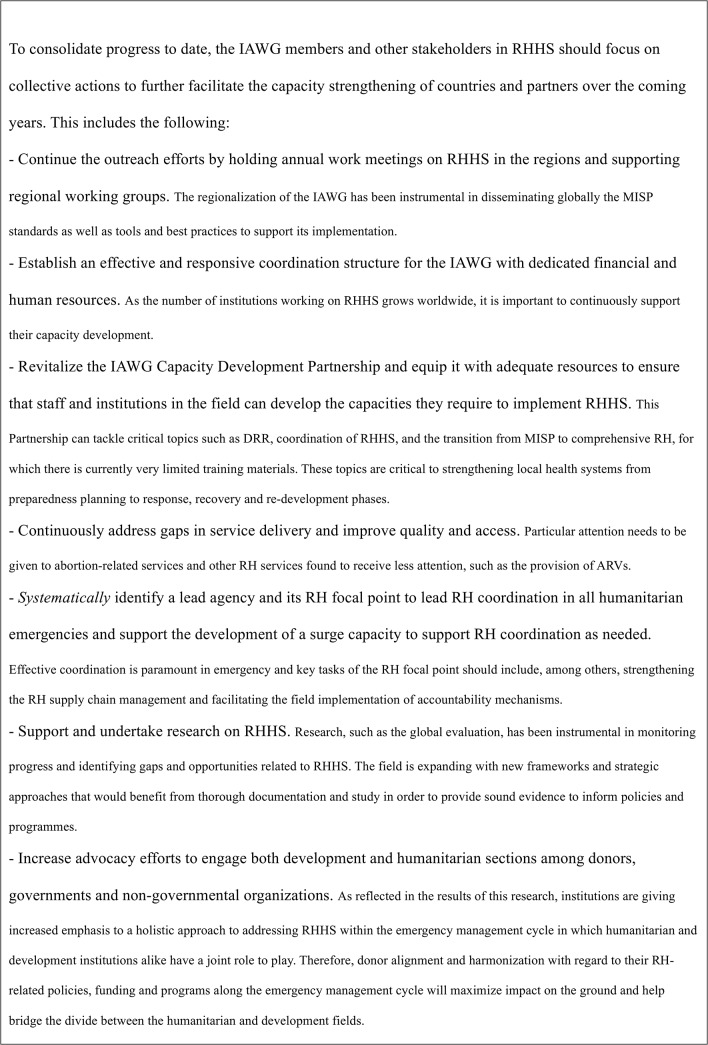
Policy and program implications of the study for the global public health community.

The results suggest growth in institutional capacity in RHHS but further research is needed to examine the nature, quality and extent of this progress. Overall, there are encouraging indications that the RHHS field may have matured. It is therefore critical to consolidate the progress to date, address identified gaps, and sustain the momentum of ongoing improvement (Fig 7).

## Supporting Information

S1 Data Set Data set on institutional capacity for reproductive health in humanitarian settings. (PDF)
