# Supplementary material for: Developing Institutional Capacity for Reproductive Health in Humanitarian Settings: A Descriptive Study
Source: PLoS One. 2015 Sep 2;10(9):e0137412. doi: 10.1371/journal.pone.0137412 (PMC4558004; doi:10.1371/journal.pone.0137412)

Institutional Capacity for Reproductive Health in Humanitarian Settings

Data set

Characteristics of institutional respondents

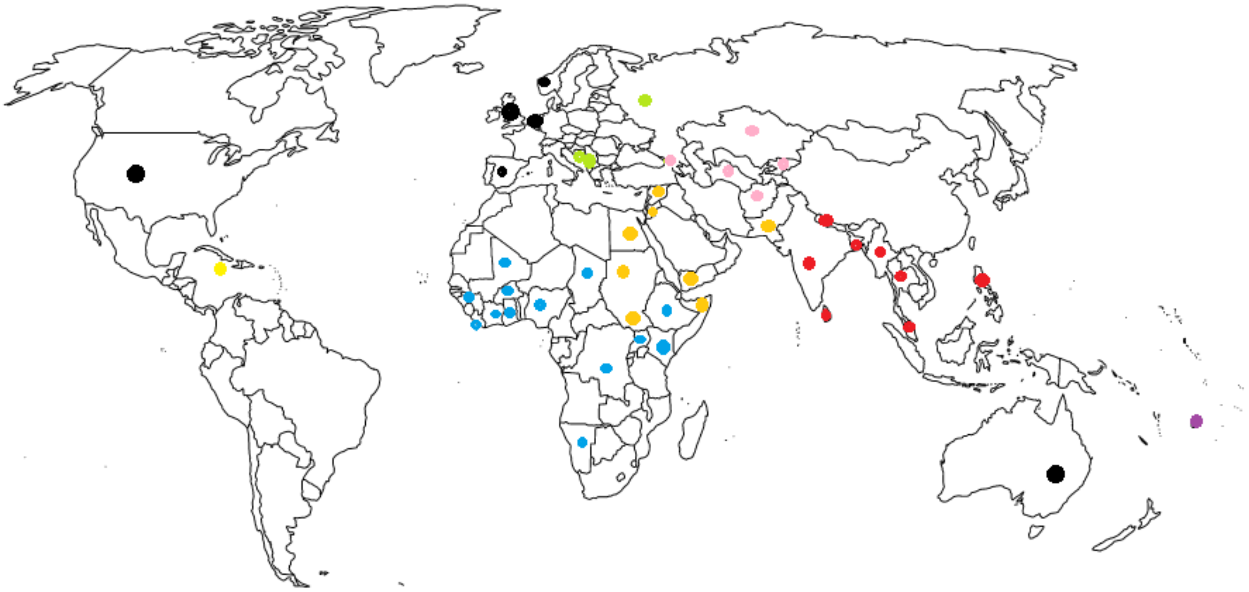

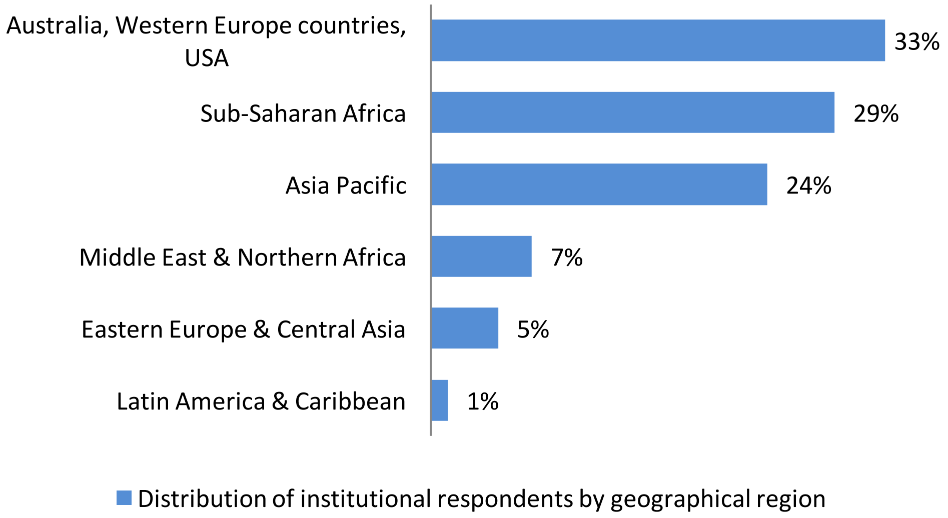

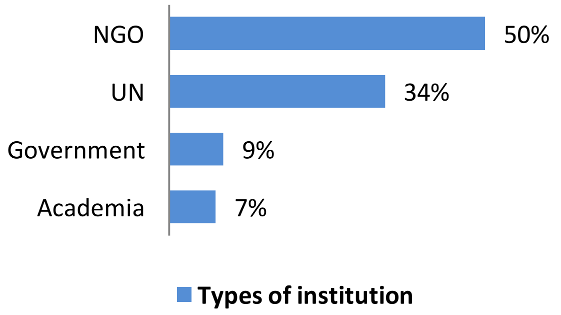
NGO
UN
Government
Academia
50%
34%
9%
7%
Types of institution

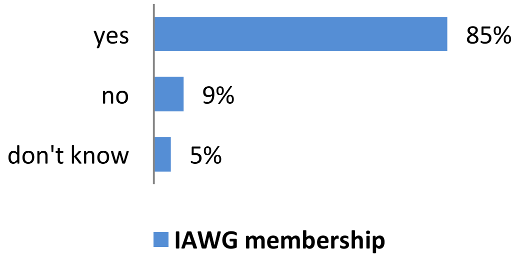
yes
no
don't know
85%
9%
5%
IAWG membership

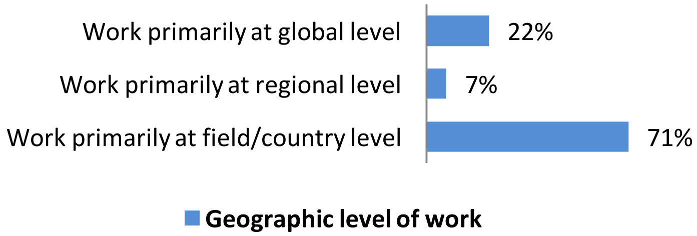
Work primarily at global level
Work primarily at regional level
Work primarily at field/country level
22%
7%
71%
Geographic level of work

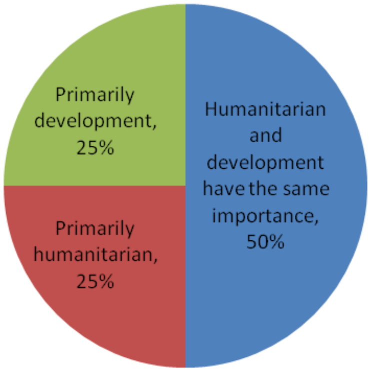
Primarily development, 25%
Primarily humanitarian, 25%
Humanitarian and development have the same importance, 50%

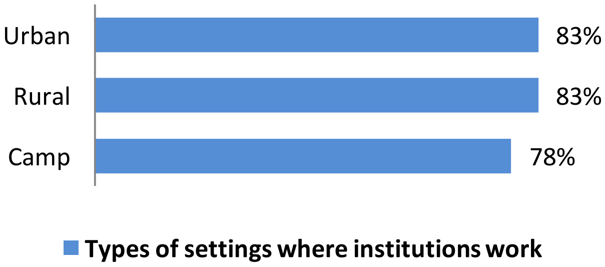
Urban
Rural
Camp
83%
83%
78%
Types of settings where institutions work

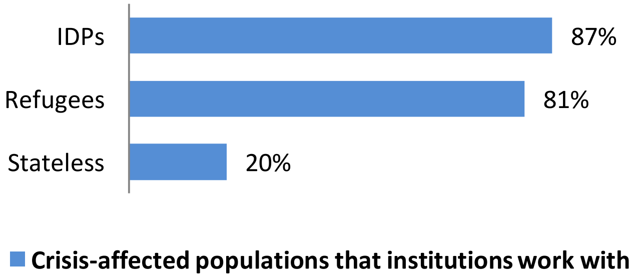
IDPs
Refugees
Stateless
87%
81%
20%
Crisis-affected populations that institutions work with

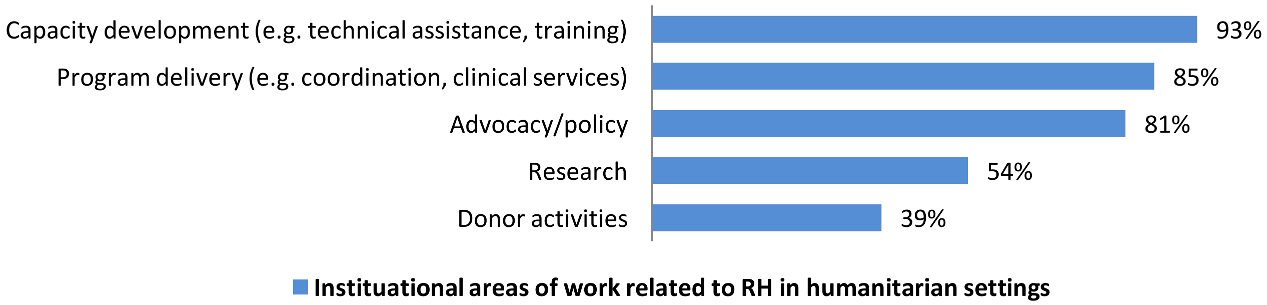
Capacity development (e.g. technical assistance, training)
Program delivery (e.g. coordination, clinical services)
Advocacy/policy
Research
Donor activities
93%
85%
81%
54%
39%
Instituational areas of work related to RH in humanitarian settings

Institutional policy

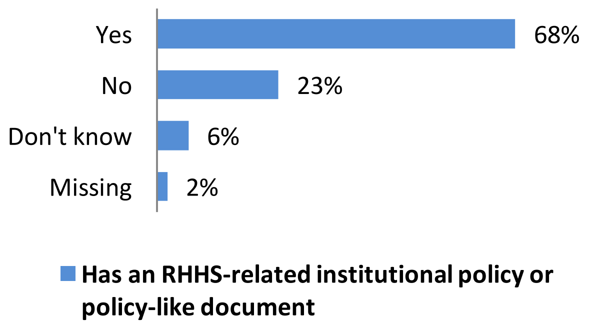

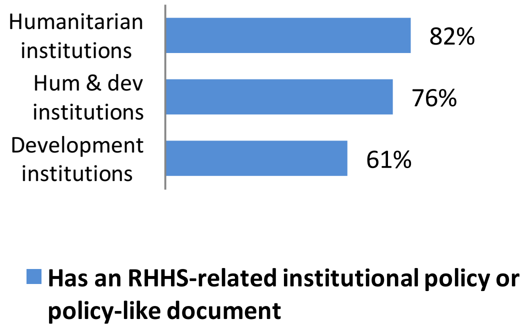

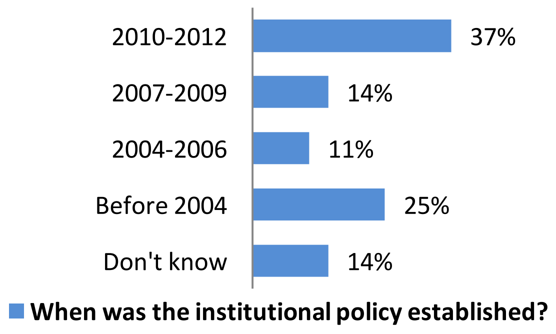

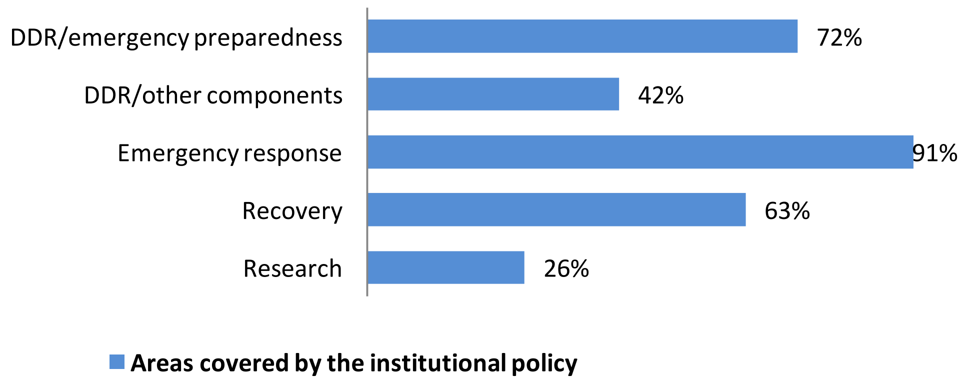

Accountability mechanisms

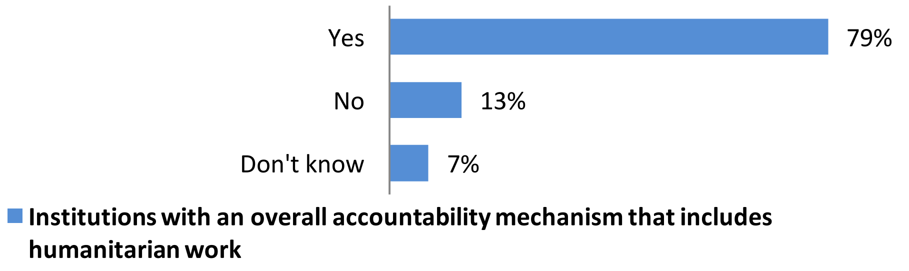

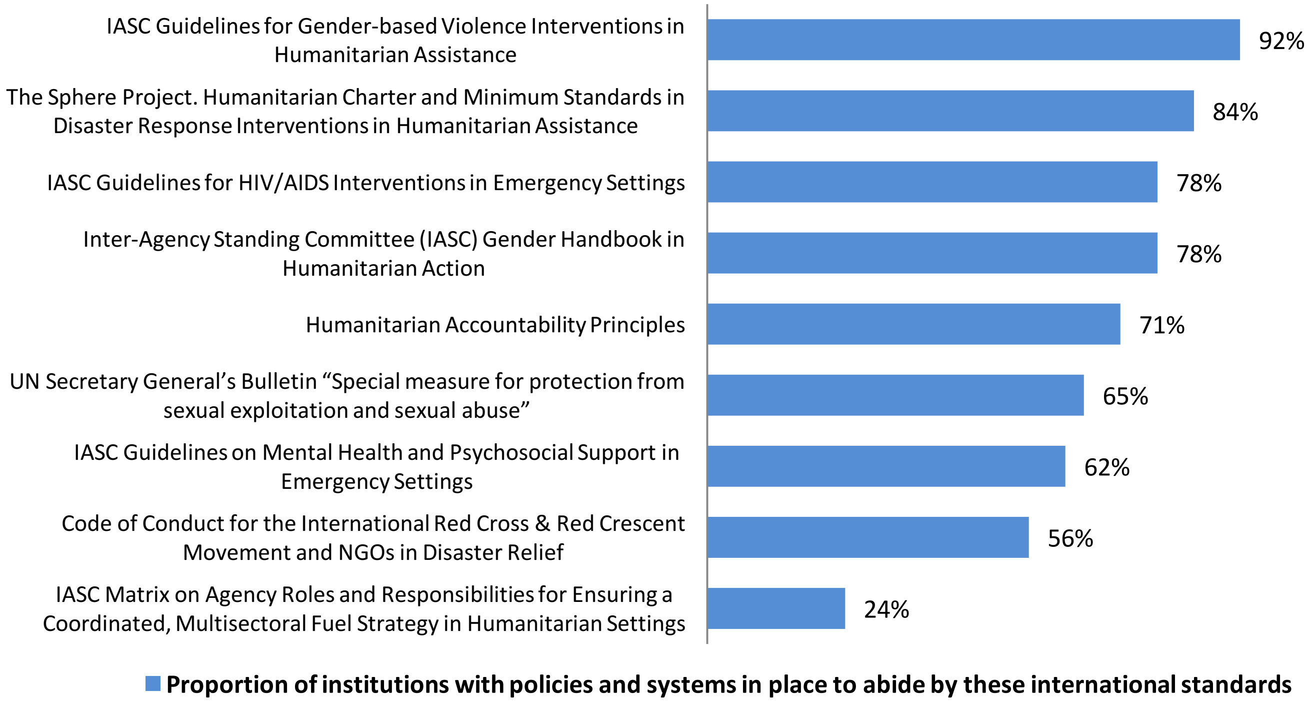

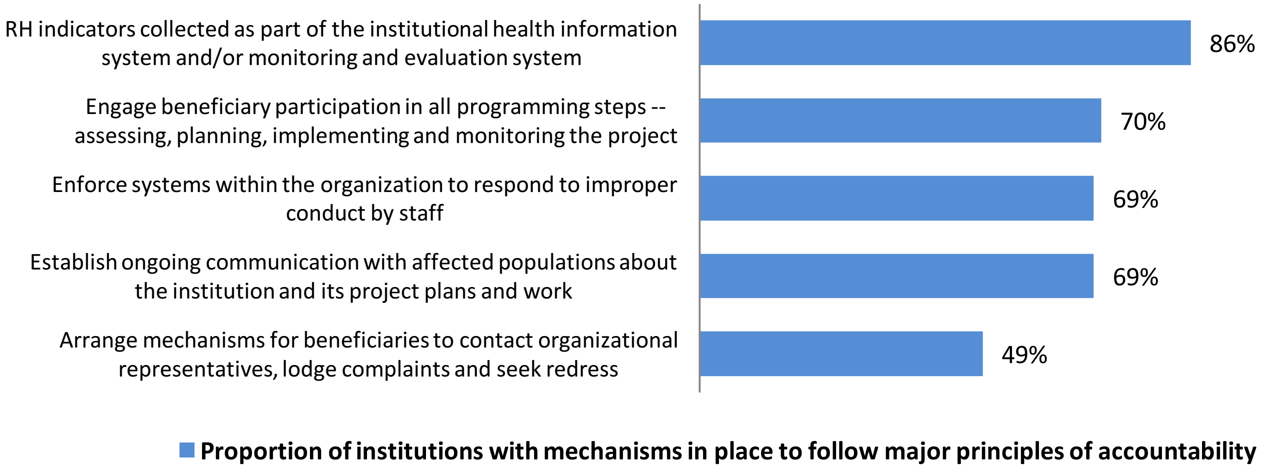

Programme delivery strategies

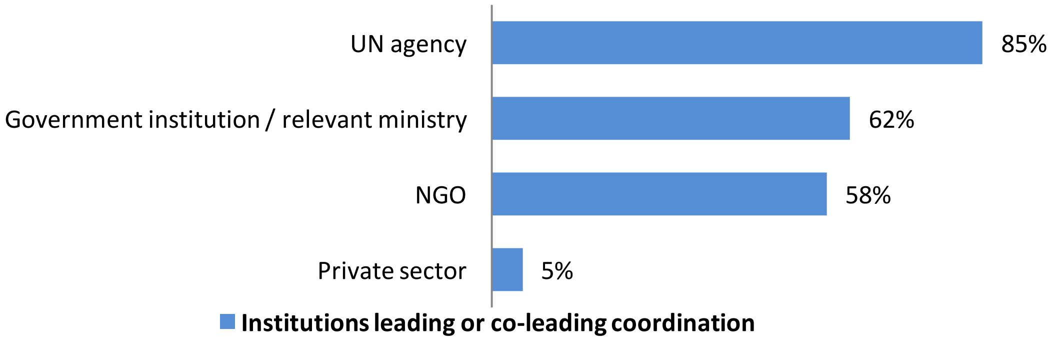

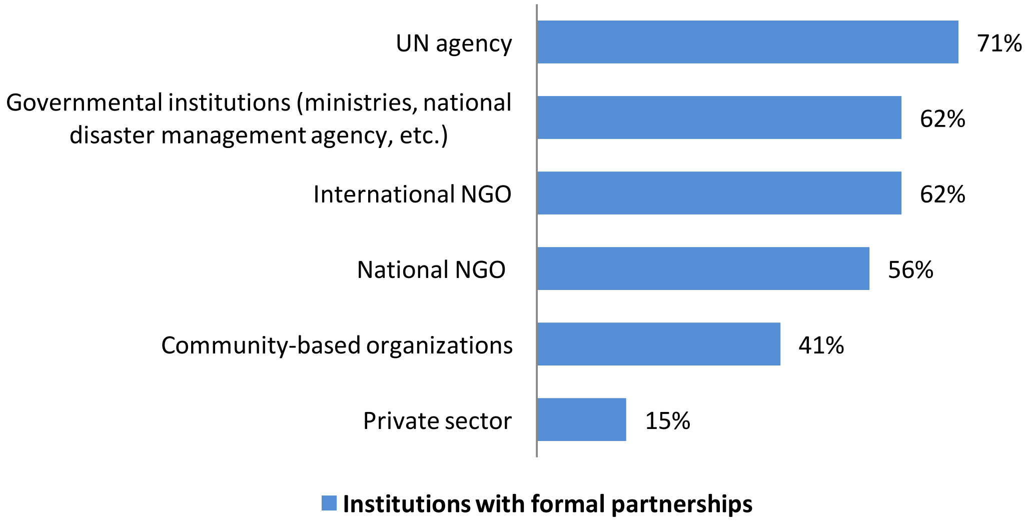

Areas of work related to RHHS

Institutional respondents reported addressing the following areas of RHHS work over the past years:

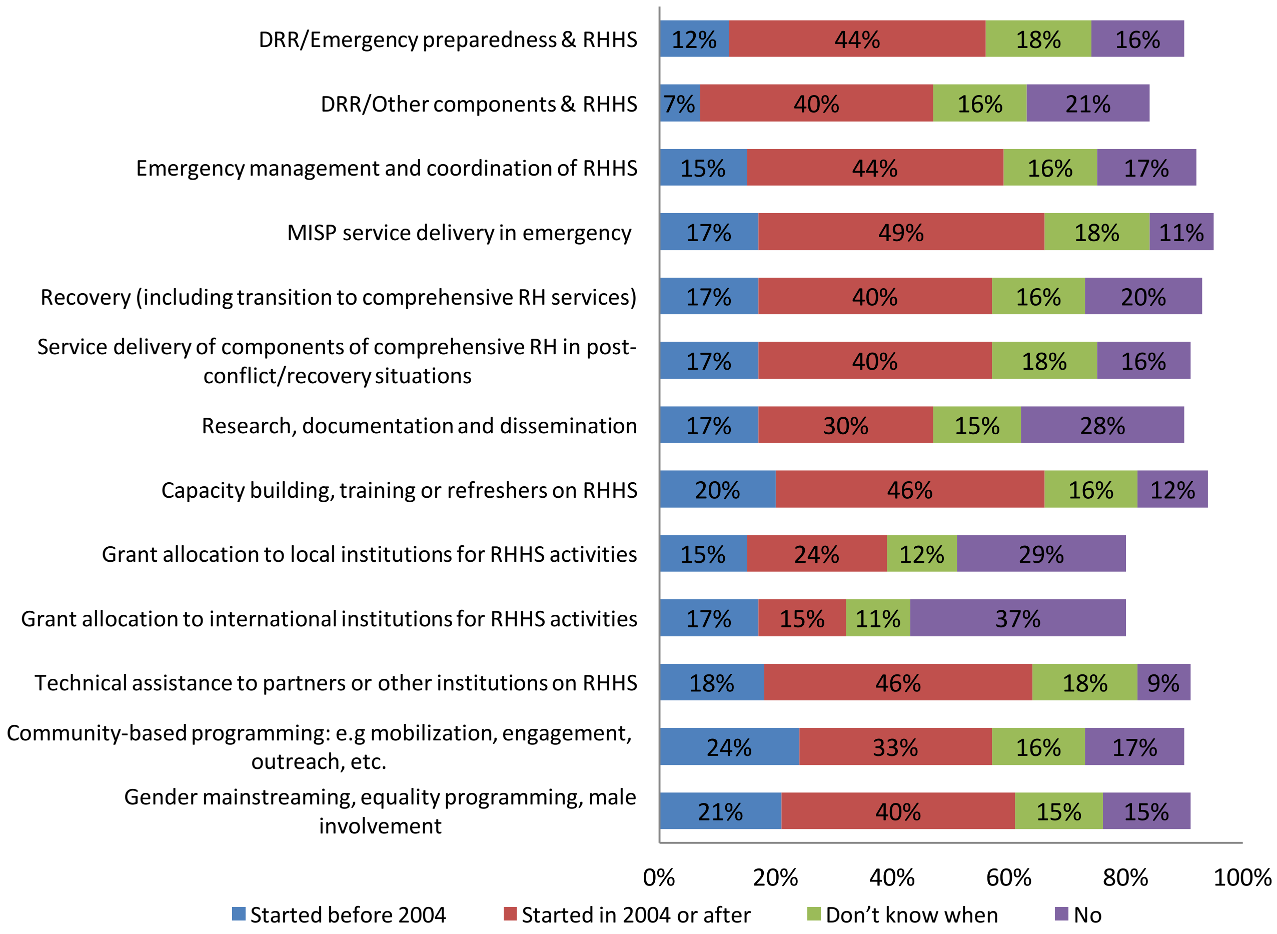

Institutional respondents reported carrying out **advocacy and policy work** to integrate RHHS into the following key areas:

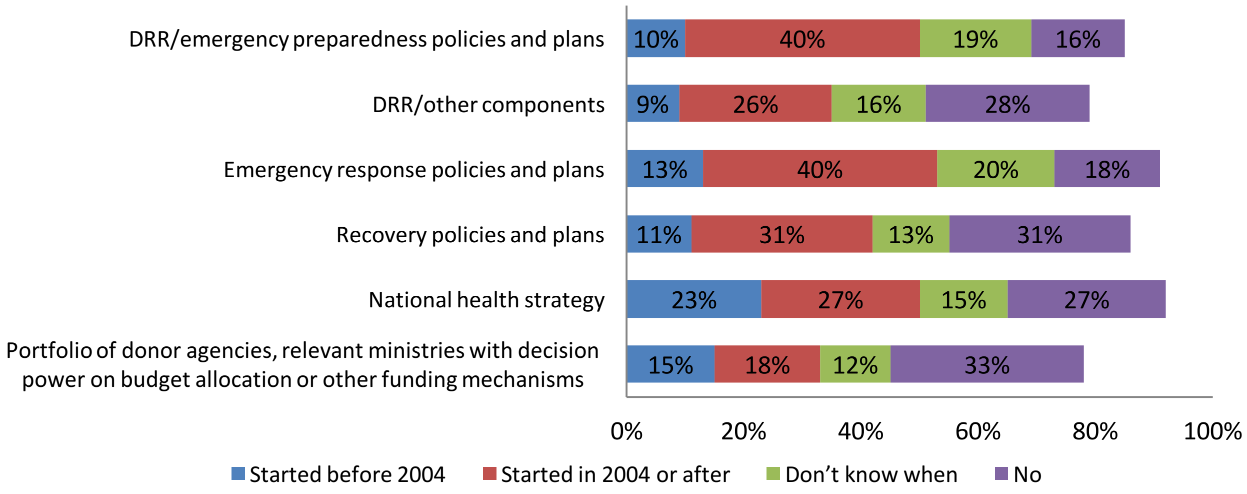

Institutional respondents reported supporting the implementation of **community-based RH services** in acute or post-acute emergency settings in the following areas of areas:

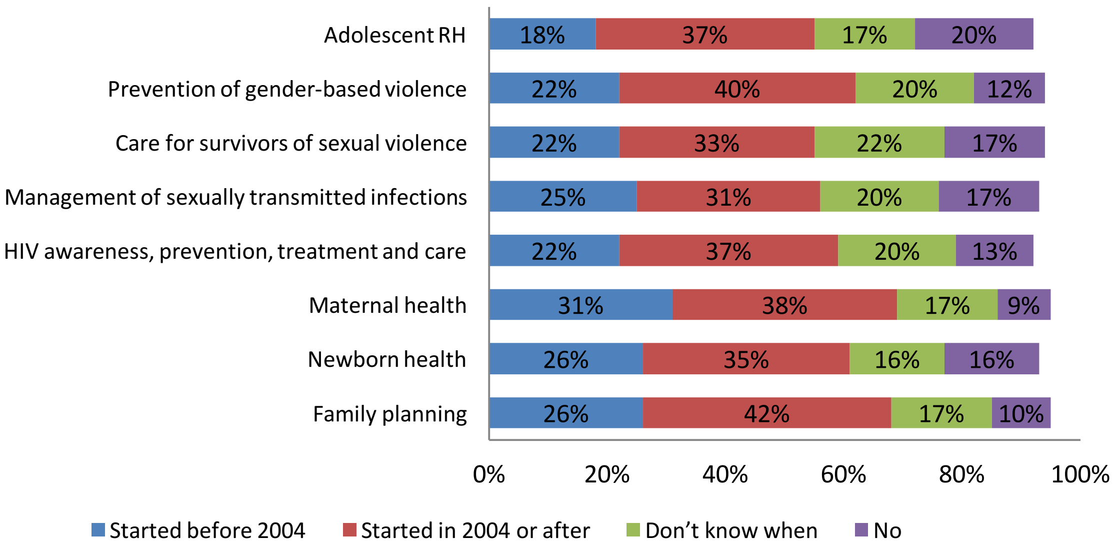

Clinical RHHS services

With regard to clinical RHHS services, institutional respondents reported having addressed the following clinical components before and since 2004 (this could be in relation to guideline development, programming, service delivery, technical assistance, advocacy, training or research):

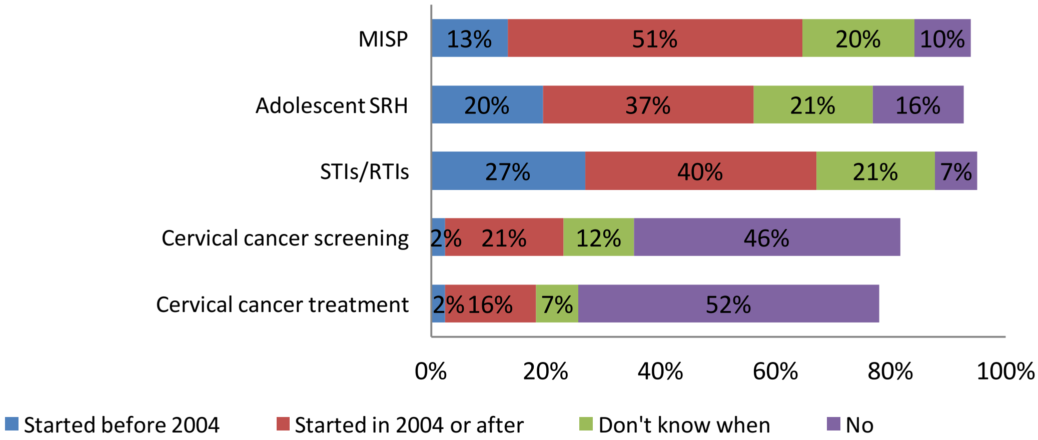

Maternal and newborn health:

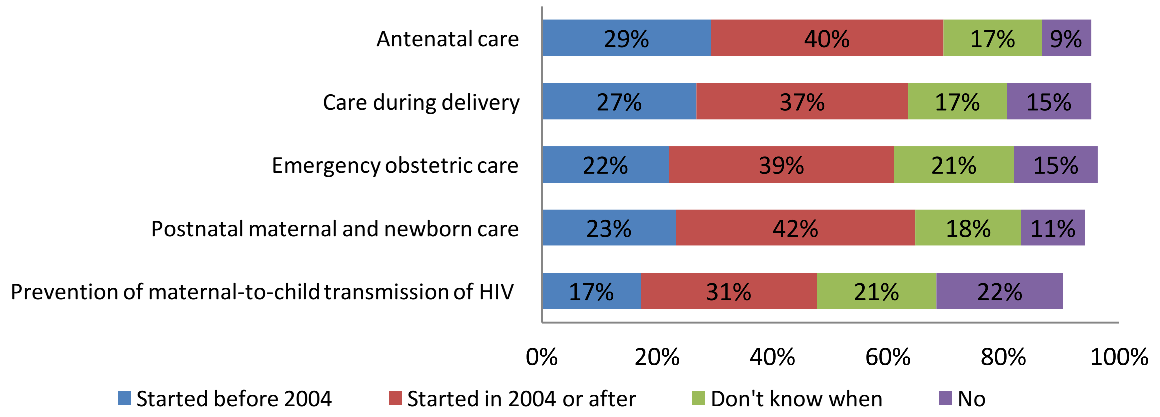

Abortion care:

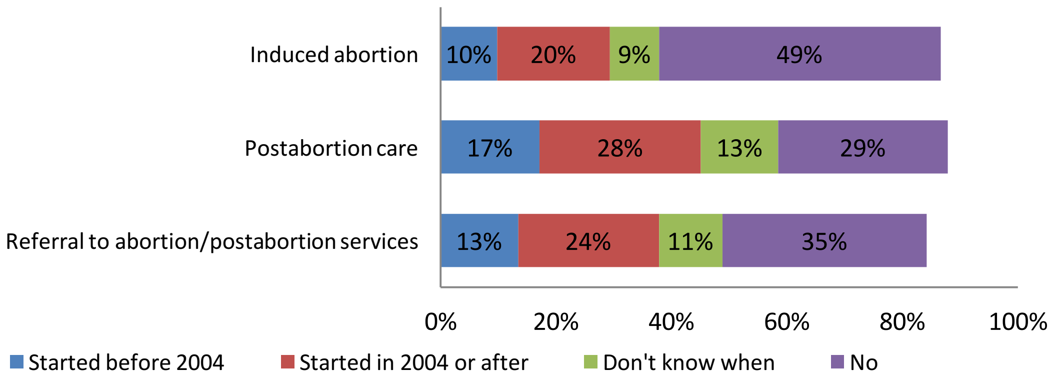

Family planning:

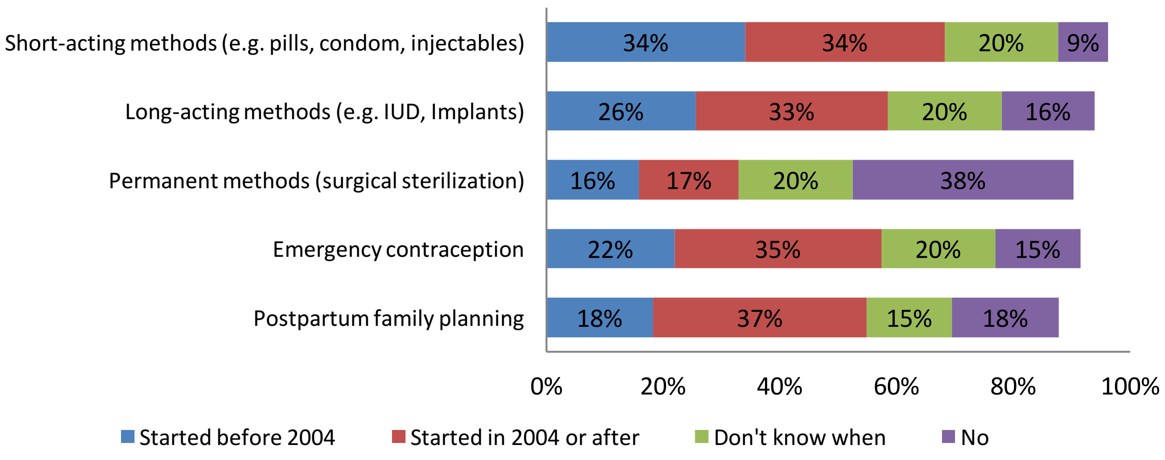

Gender-based violence:

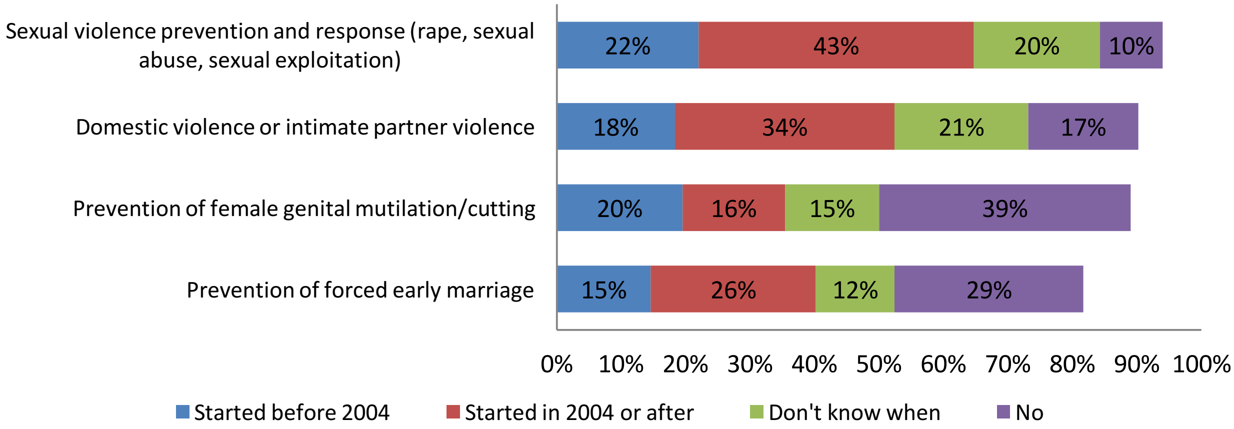

HIV:

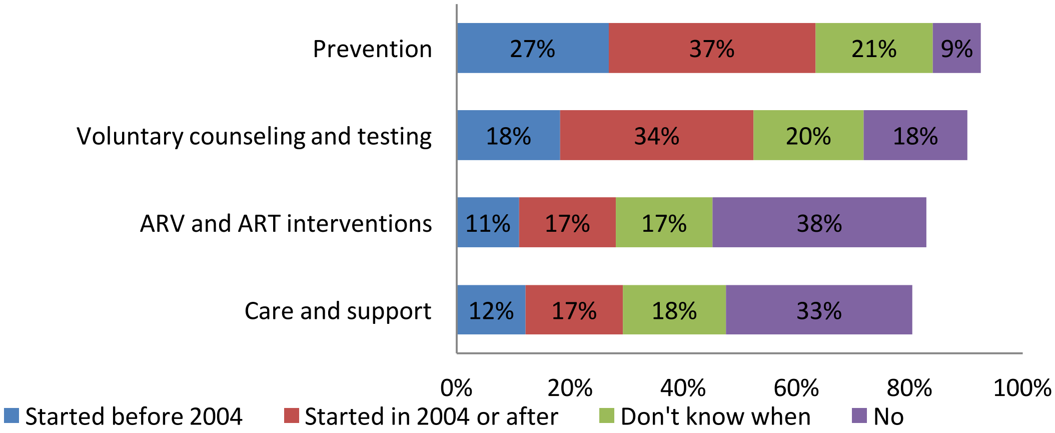

Financial resources

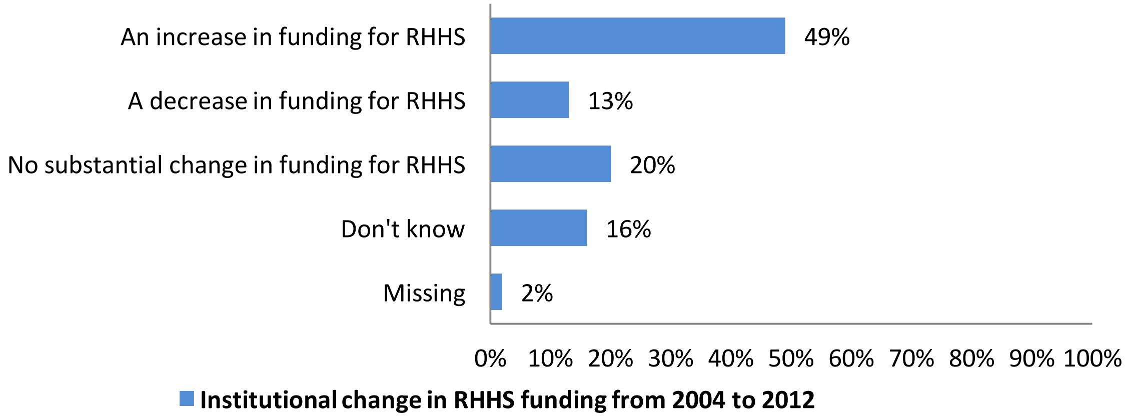

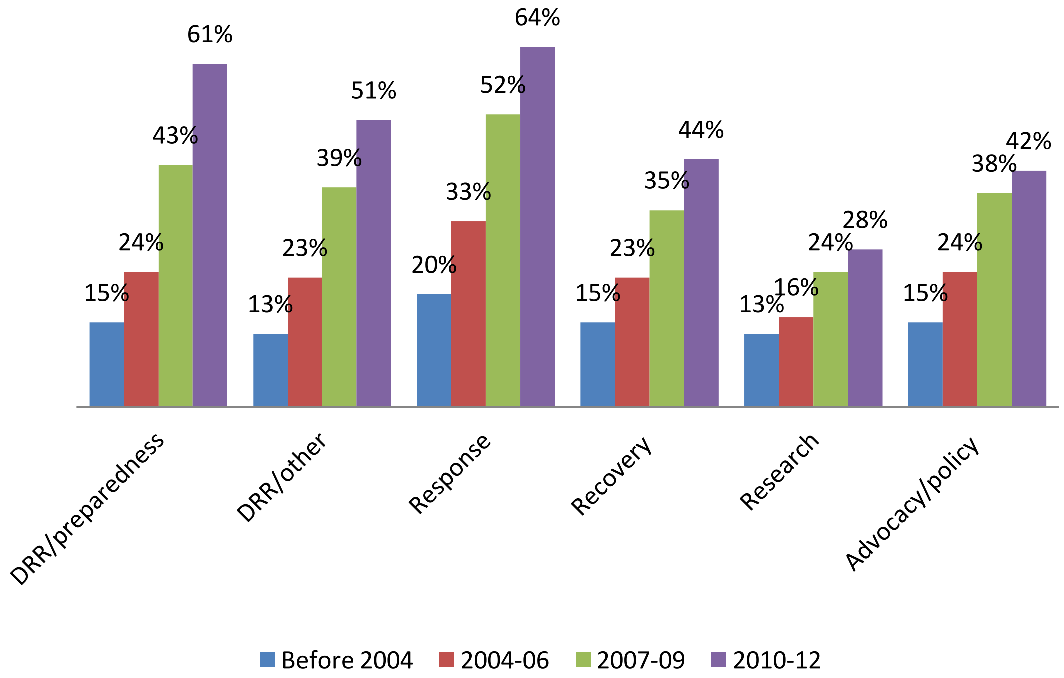

Dedicated budget for RHHS by areas of work and time period

Human resources

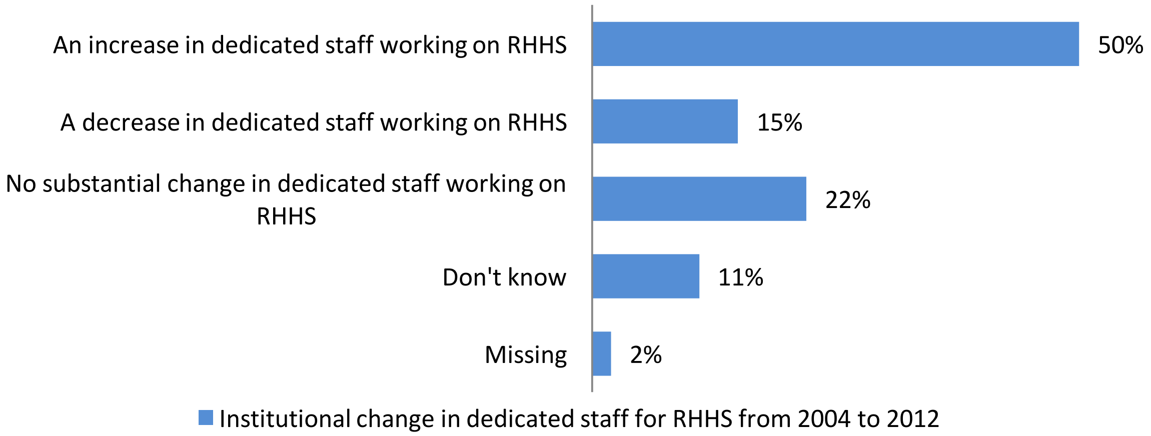

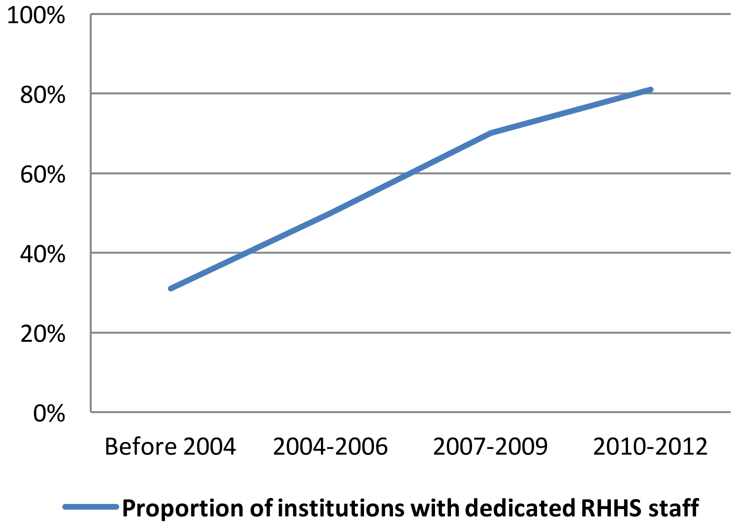

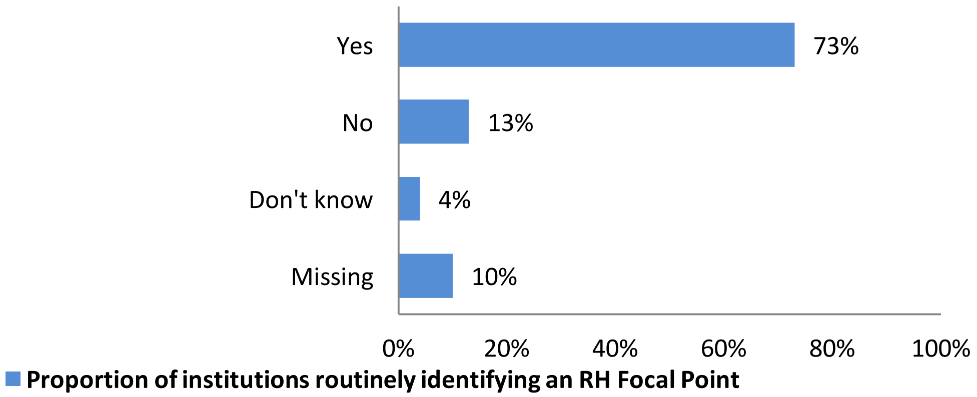

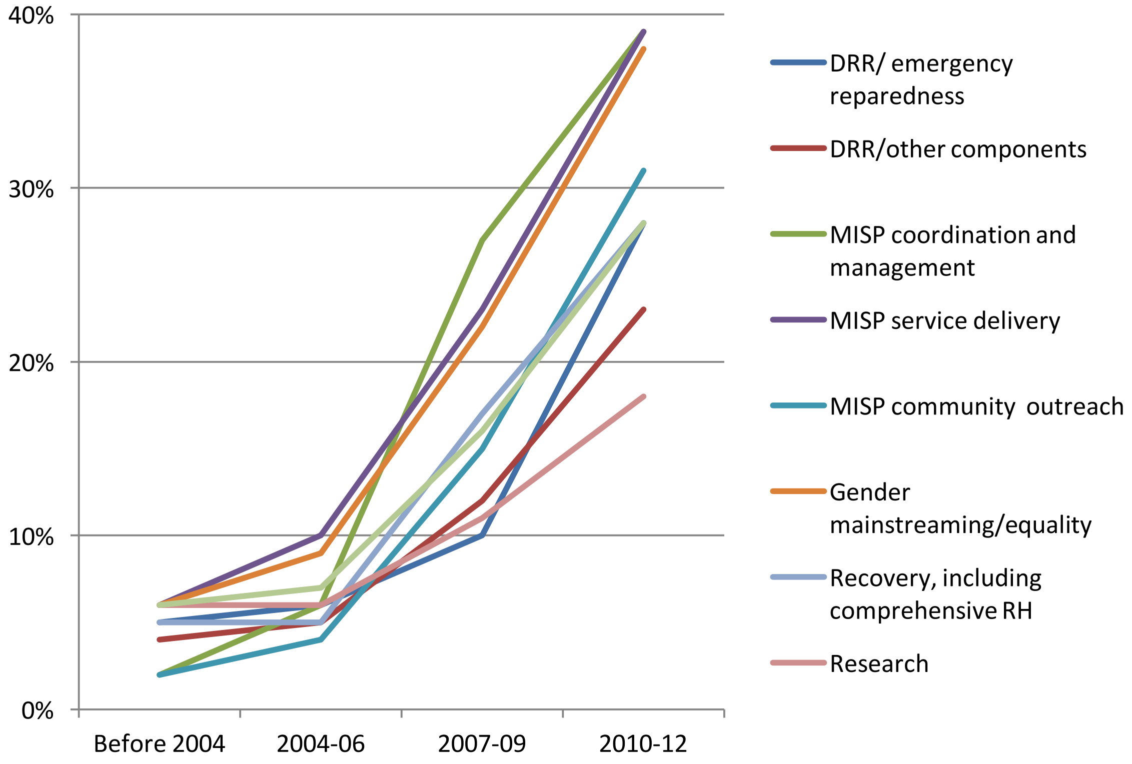

Proportion of institutions reporting high-level workforce competencies in different RHHS areas

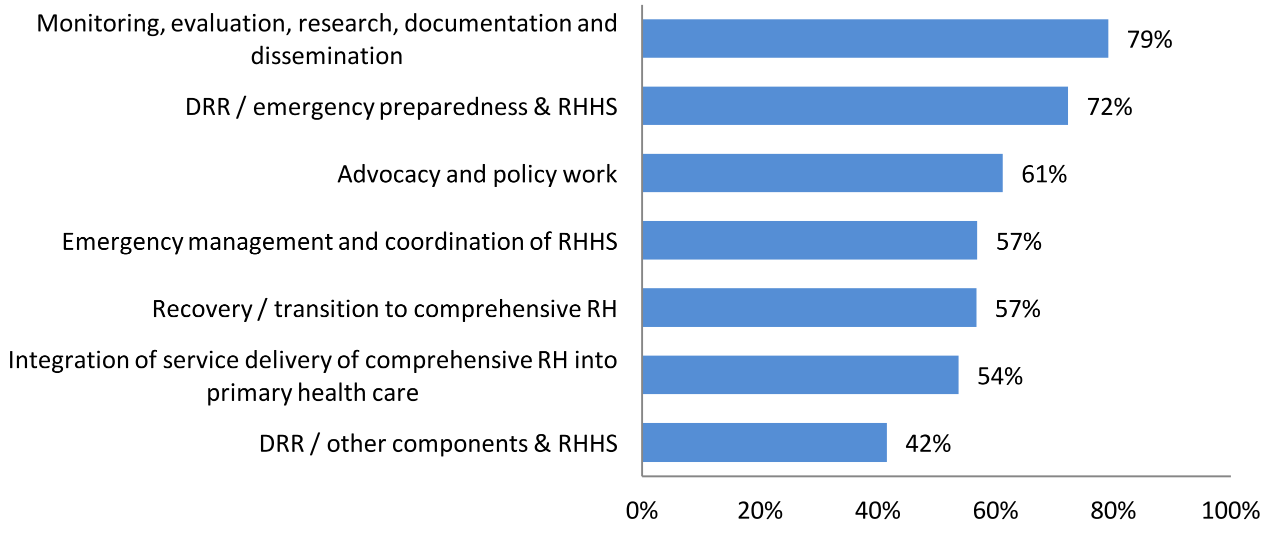

Proportion of institutions requiring programmatic guidance to be developed by IAWG to support their institution's work on RHHS

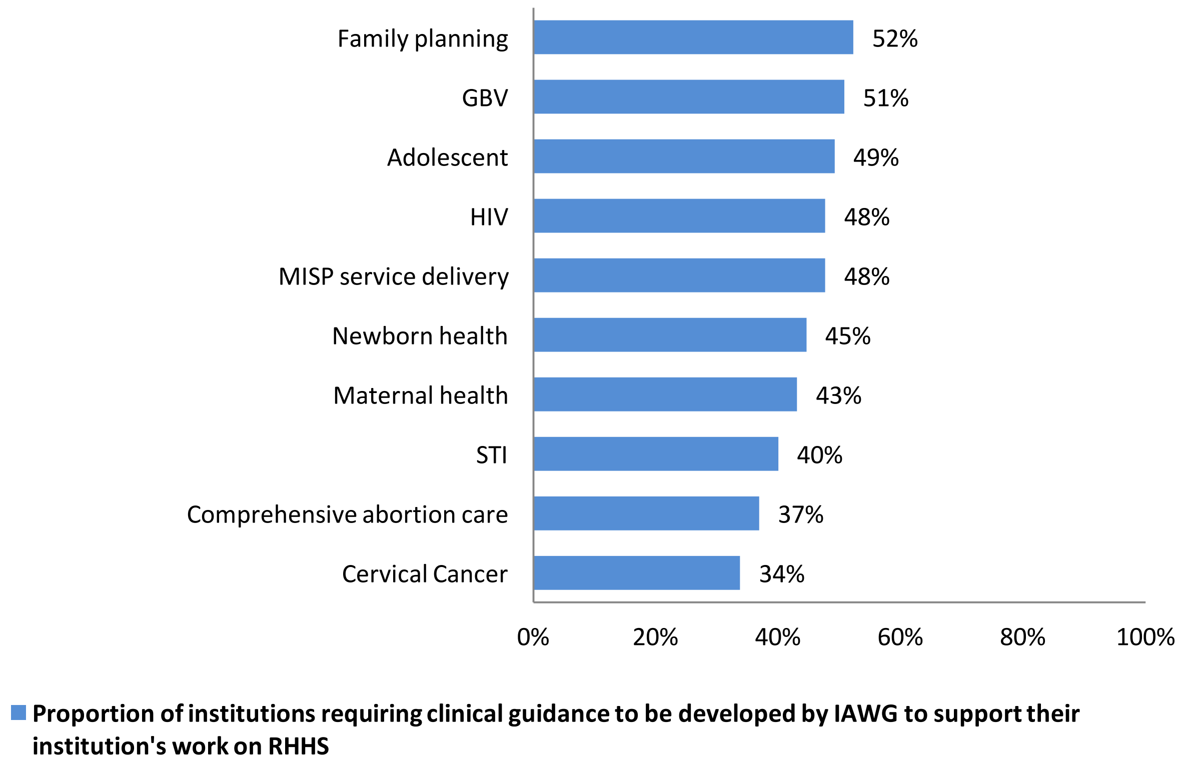
Family planning
52%
GBV
51%
Adolescent
49%
HIV
48%
MISP service delivery
48%
Newborn health
45%
Maternal health
43%
STI
40%
Comprehensive abortion care
37%
Cervical Cancer
34%
0%
20%
40%
60%
80%
100%
Proportion of institutions requiring clinical guidance to be developed by IAWG to support their institution's work on RHHS

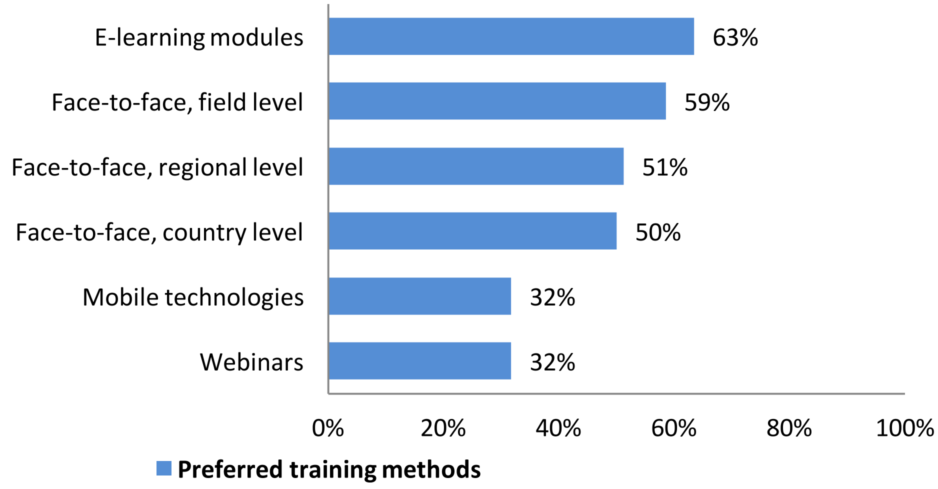
E-learning modules
63%
Face-to-face, field level
59%
Face-to-face, regional level
51%
Face-to-face, country level
50%
Mobile technologies
32%
Webinars
32%
0%
20%
40%
60%
80%
100%
Preferred training methods

Material resources

Guidance

Institutional respondents were asked to rank the top five most useful resource materials among 21 key RHHS documents published or updated between 2004 and 2012. The results are shown in the table below. Note that all 21 publications received votes.

Top five and top 10 most useful resource materials since 2004

1. Inter-agency Working Group on Reproductive Health in Crises: *Inter-agency Field Manual on Reproductive Health in Humanitarian Settings.* Geneva; 1999, 2010 revision (name of the first edition: Reproductive Health in Refugee Situations; an Inter-agency Field Manual, IAWG, 1999)
2. IPPF/Women's Refugee Commission: *Synopsis of the MISP ("Cheat Sheet").* New York; 2008, revised 2009.
3. United Nations Population Fund: *Interagency Reproductive Health Kits*. Geneva; various editions since 2004.
4. Women's Refugee Commission: *MISP Distance Learning Module*. New York; 2007, revised 2011.
5. International Planned Parenthood Federation, United Nations Population Fund, University of New South Wales: *SPRINT Facilitator's Manual on SRH Coordination in Crises*. Kuala Lumpur; 2009.

---

6. The Sphere Project: *Humanitarian Charter and Minimum Standards in Disaster Response*. Geneva; 2000, 2011.
7. Inter-Agency Standing Committee (IASC*): Guidelines for GBV interventions in humanitarian emergencies*. Geneva; 2005.
8. Save the Children/UNFPA: *Adolescent Sexual and Reproductive Health Toolkit for Humanitarian Settings*. New York; 2009.
9. The International Rescue Committee: *Clinical Care for Sexual Assault Survivors: A Multi-Media Training Tool*. New York; 2008.
10. Gender-based Violence Area of Responsibility Working Group: *Handbook for Gender-based Violence Interventions in Humanitarian Settings*. [No location]; 2010.

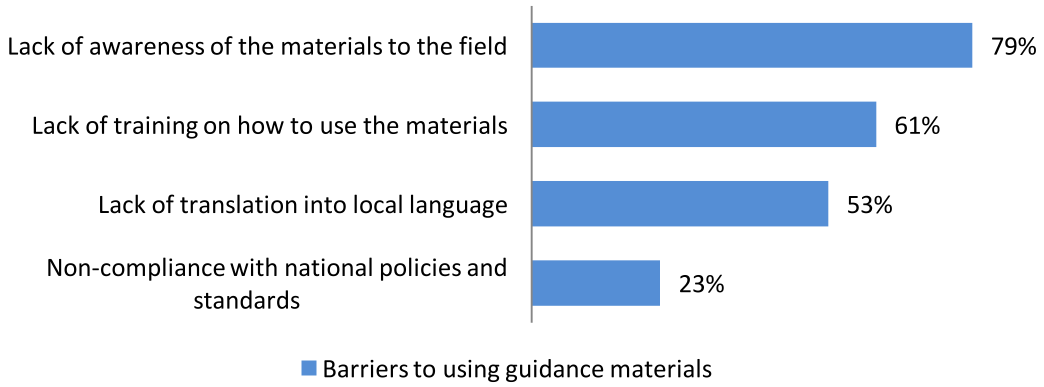

RHHS commodities

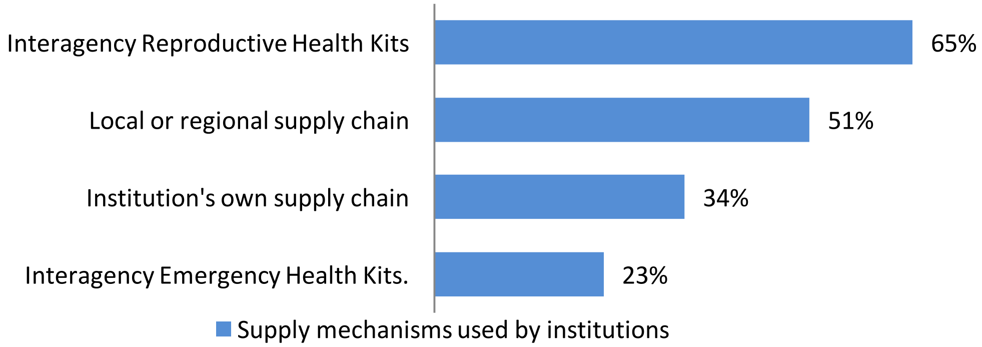

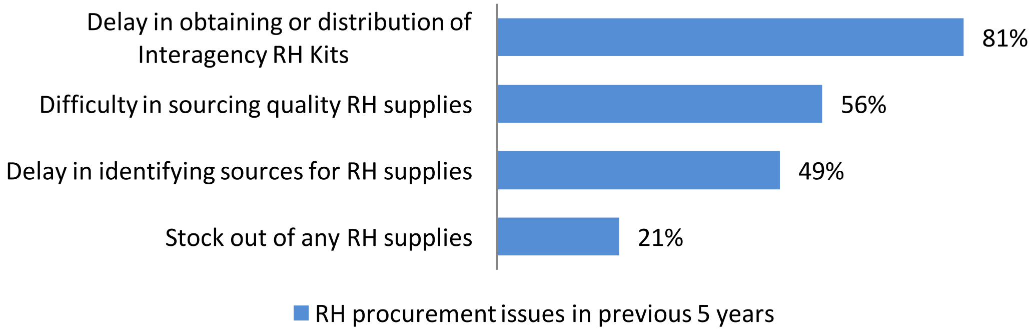

Supplement: S1 Data Set — (PDF) [file pone.0137412.s001.pdf]
